# Methodological insights from the EPISTOP trial to designing clinical trials in rare diseases—A secondary analysis of a randomized clinical trial

**DOI:** 10.1371/journal.pone.0312936

**Published:** 2024-12-03

**Authors:** Stephanie Wied, Ralf-Dieter Hilgers, Nicole Heussen, Katarzyna Kotulska, Maya Dirani, Mathieu Kuchenbuch, Sergiusz Jozwiak, Rima Nabbout

**Affiliations:** 1 Institute of Medical Statistics, RWTH Aachen University, Aachen, Germany; 2 Medical School, Sigmund Freud Private University, Vienna, Austria; 3 Department of Neurology and Epileptology, The Children’s Memorial Health Insitute, Member of Epicare, Warsaw, Poland; 4 Department of Pediatric Neurology, Imagine Institute Paris, Necker-Enfants Maelades Hospital, Reference Centre for Rare Epilepsies, Member of Epicare, University Paris cite, Paris, France; 5 Research Department, The Children’s Memorial Health Insitute, Member of Epicare, Warsaw, Poland; Belgrade University Faculty of Medicine, SERBIA

## Abstract

**Background:**

In clinical research, the most appropriate way to assess the effect of an intervention is to conduct a randomized controlled trial (RCT). In the field of rare diseases, conducting an RCT is challenging, resulting in a low rate of clinical trials, with a high frequency of early termination and unpublished trials. The aim of the EPISTOP trial was to compare outcomes in infants with tuberous sclerosis (TSC) who received vigabatrin preventively before the seizures onset with those who received it conventionally after. The study was designed as a prospective, multicentre, randomized clinical trial. However, ethics committees at four centres did not approve this RCT design, resulting in an open-label trial (OLT) in these four centres and an RCT in the other six centres. In this paper, we re-analyse the data from the EPISTOP trial using methods to investigate the influence of allocation bias on the results of the EPISTOP trial.

**Method:**

A bias-corrected analysis is used to support and strengthen the published results. We included a term representing the effect of selection bias as an influencing factor on the corresponding endpoint in the statistical model. Thus, the treatment effect estimates for the primary endpoint of time to first seizure and additional secondary endpoints are adjusted for the bias effect.

**Result:**

The bias-corrected analyses for the primary endpoint show that the estimated hazard ratio and associated confidence intervals are in a very similar range (original analysis: HR 2.91, 95%-CI [1.11 to 7.67], p-value 0.0306; bias-corrected analysis: HR 2.89, 95%-CI [1.10 to 7.58], p-value 0.0316). This was also the case for the secondary endpoints.

**Conclusion:**

The statistical re-analysis of the raw trial data therefore supports the published results and confirms that there is no additional bias introduced by randomization, thereby increasing the value of the results. However, this highlights that this aspect needs to be considered in future trials, especially in rare diseases, to avoid additional biases in an already small sample size where it may be difficult to reach significance.

## Introduction

Rare diseases are defined in the EU as diseases occurring in less than 1 per 2.000 individuals in the general population [1], and in the US as conditions that affect fewer than 200.000 individuals throughout the country [2]. Although they are called “rare,” more than 8.000 rare diseases have been actually identified [3], affecting an estimated 350 million individuals globally, i.e. ≈5% of the world’s population. The limited prevalence of these diseases creates, *de facto*, a significant challenge in conducting clinical trials. Some key obstacles include the limited knowledge about the natural history and pathophysiology of the diseases, the heterogeneity and the variability of the phenotypes, the lack of biomarkers, the limited pool of eligible individuals, the lack of meaningful and patient centered end points and the difficulties inherent in conducting multinational trials [4–7]. This probably explains the low rate of clinical trials in rare diseases, and the high frequency of early termination and unpublished trials [8, 9]. Consequently, only 11.5% of the clinical trials registered on clinicaltrials.gov focus on rare diseases [8] with an estimated 30.2% and 31.5% of these trials remaining uncompleted or unpublished at 4 years, respectively [9]. This low number of clinical trials has important repercussions for affected individuals and their caregivers: less than 10% of rare diseases have treatments available, and only 22% of rare diseases have already been the subject of a clinical trial [10, 11].

In addition, clinical trials on rare diseases differ from those on non-rare diseases not only in term of small number of patients per trial but also employ less robust methodologies. Typically, these trials adopt a single-arm, non-randomized and open-label design. The statistical analyses of trials involving small populations poses challenges, including an increased risk of bias, limited statistical power, constraints on the use of traditional statistical methods [5, 6, 12]. Moreover, the use of standard classical methodologies, not tailored specifically to rare diseases, leads to a loss of power to show positive effects as well as insufficient capacity to address all the questions that patients, clinicians, and regulatory agencies need to obtain from trials. Consequently, conducting trials with a high level of evidence, as randomized controlled trials, including placebo-control arm, is particularly challenging to conduct in the context of rare diseases. This underscores the urgent need to address this issue and explore alternative trial design options when studying treatments for rare diseases in order to maximize the use of available data and optimize the reliability and validity of study results [12, 13]. To tackle the particular challenges in statistical design and analysis of small population group trials, methodologists considered directions for new developments in this area [12]. The three FP7-funded projects, IDeAl [14], asterix [15] and InSPiRe [16] have addressed some of those challenges and generated numerous innovative statistical methods for small population clinical trials. While the developments reached in those projects were exceptional, they lacked practical validation in real-world settings. The EJP RD WP20 initiative has built on the advancements made in these three projects to pursue further growth and achieve additional milestones.

The EPISTOP clinical trial is a prime example of the challenges related to clinical trials for rare disease. This trial followed initial work identifying epileptiform activity captured on video-EEG prior to seizure onset as biomarker of susceptibility to further epilepsy in individuals with tuberous sclerosis (TSC) [17, 18]. In animal models, preventive treatment initiated before seizures onset significantly decreased the risk of epilepsy as well as associated comorbidities [19, 20]. The objective of EPISTOP was to compare the outcome in TSC infants receiving vigabatrin, an antiseizure medication (ASM), in a preventive way, based on EEG monitoring and before the seizures’ onset, versus receiving it in a conventional way, after seizure onset. The study was designed as a prospective randomized clinical trial and involved ten centers, with nine in the Europe and one in Australia [21]. However, ethical committees at four sites did not approve this RCT design. Among these, two sites deemed only preventive treatments acceptable, whereas the remaining two sites considered conventional treatment as the sole acceptable option. The principal investigators then decided to conduct an open-label trial (OLT) in these four centers, in parallel with the randomized trial in the other six centers. The study was able to include 94 individuals with exclusion of 40 patients and remaining with 54 of the 100 expected initially. Finally, 27 entered the RCT and 27 the OLT. The time to the first clinical seizure, the primary endpoint, was significantly longer in preventive than conventional arm in RCT (614 (95% confidence interval = 364–infinity) days vs 124 (118–215) days), in OLT (602 (403-infinity) vs 124 (78–242) days) and in pooled analysis (614 (474-infinity) days versus 124 (114–200) days). Secondary endpoints in this study allowed confirming the benefit of a preventive approach for epilepsy in individuals with TSC. Indeed, the pooled analysis showed that preventive treatment reduced at 24 months the risk of clinical seizures (odds ratio [OR] = 0.21 (95% CI: 0.05–0.9), p = 0.032), drug-resistant epilepsy (OR = 0.23 (0.06–0.83), p = 0.022), and infantile epileptic spasms syndrome (OR = 0 (0–0.33), p = 0.001). However, the EPISTOP consortium noticed various effects in the data that could not be confirmed due to a gap in applying standard statistical analysis methodologies for finite populations.

In this paper, we aimed to reanalyze the data of the EPISTOP trial applying methodologies that might overcome some bias. With this example, we propose the critical points to be considered in the preparation of clinical trials in rare diseases, using the most appropriate methods for each gap or challenge.

## Materials and methods

### Study design and randomization

This is a non-preplanned secondary analysis of the randomized, open label, multicenter two arm parallel group EPISTOP trial with an 1:1 allocation ratio to investigate whether bias may impact the study results. The study design and results are presented elsewhere [21]. The original study was registered in ClinicalTrials.gov database, under number NCT02098759, approved by the Bioethics Commitee at the Children’s Memorial Health Institure (IPCZD) in Warsaw, Poland (66/KBE/2013). All additional relevant national/local authorities conducted the trial in accordance with the Declaration of Helsinki, and all patients or their legal surrogates provided informed consent. Additional details on approvals and the informed consent procedure are available in the primary trial publication [21]. In brief the EPISTOP trial is an open label randomized multicentre trial, with a two-arm parallel group design without any interim analysis of adaptation to prove the superiority hypothesis of a preventive versus a conventional group with regard to the onset of clinical seizures in children. Initially, it was planned to randomize patients in all participating centers, but the RCT was not approved at 4 out of 10 sites, and therefore, subjects at these sites were enrolled in a parallel open-label trial. For conventional treatment, vigabatrin was administered after the occurrence of electrographic or clinical seizures. For preventive treatment, vigabatrin was administered if epiletiform activity was detected on electroencephalography before the onset of seizures. The recruitment period started on 01/03/2014 and ended on 10/01/2017. Randomization was performed centrally stratified for study sites. We use the actual treatment administration in consecutive patient enrolment order to estimate the bias effect. With this, we will use bias corrected analysis models for time to event [22] and continuous outcomes [23] in two arm parallel group trials to perform the reanalysis.

### Definition of endpoints

#### Time to first seizure

The primary study endpoint is the time-to-event outcome. The event in this case is a witnessed seizure by the family, the physician or a recorded seizure on video-EEG. This primary endpoint was defined by the time from birth to the onset of the first event. It was calculated by days and can be considered as an objective measure. Within a sensitivity analysis, the time from study entry to the first clinical seizure is additionally considered in this article.

#### Bayley Scales of Infant Development (BSID)

One of the investigated secondary endpoints was the neurodevelopmental delay at the age of two years. This neurodevelopmental delay is assessed using the continuous BSID-III cognitive score (Bayley Scales of Infant Development, 3rd edition) which is an assessment tool for diagnosing developmental delays in early childhood. In addition, the subcomponents of the BSID-III cognitive score are considered as further endpoints. Indeed, the Bayley-III assessment divides its scores into three main composite scores: cognitive (BSID Cognitive), language (BSID Language), and motor (BSID Motor). Additionally, it incorporates primary caregiver-reported questionnaires to evaluate Social-Emotional (BSID SE) and Adaptive Behavior (BSID GAC) aspects. The average range for these scores is 100±15. A moderate delay is detected when the score falls below 70 (< −2 DS), and a severe delay is indicated when it’s below 55 (< −3 DS). The Language Scale originates from two subtests: the Receptive Communication subtest (BSID R. Language) and the Expressive Communication subtest (BSID E. Language). Similarly, the Motor Scale originates from two subtests: the Fine Motor subtest (BSID Fine Motor) and the Gross Motor subtest (BSID Gross Motor). The average score for these subtests were 10±3 with a delay defined as a score bellow 4 (< −2 SD). These scores are the gold standard for the assessment of neurodevelopmental outcome.

### Statistical analysis

In this reanalysis, the influence of selection bias on the study results of the EPISTOP study is examined. A bias-corrected analysis will be used to support and reinforce the published results. In addition, a bias-adjusted treatment effect between the preventive and the conventional treatment will be calculated. The bias correction is performed by including a term representing the selection bias effect as an influence factor on the corresponding endpoint in the statistical model. Thus, the treatment effect is estimated adjusted for the bias effect. The randomization sequence was recreated by the implemented allocation to treatment in consecutive order, i.e. allocation sequence, due to missing information and details on the underlying randomization procedure. Since the trial is open-label, potential assignments could be reordered, leading to guessing the next drug to be assigned. Assuming that the randomization procedure tends to produce balanced treatment allocations allocation bias may influence the study results, by allocation of patients with better prognosis to the favored treatment. This is mirrored by the bias effect *τ*. In the first step, the assignment is noted for the 35 randomized patients of the study in consecutive order. Then, for each randomized patient, the number of previously assigned patients to the conventional treatment (*N*_*C*_(*i* − 1)) and to the preventive treatment (*N*_*P*_(*i* − 1)) is assessed. This information enables an easy identification of balancing of the allocation in the time course (see Table 1). Due to drop outs before starting the study, a total of 27 of the 35 patients were actually included in the final analysis of this study. Nevertheless, the allocation reproduction was based on all 35 patients randomized to prevent loss of information in the allocation order. The statistical analyses described in the following were perfomed using SAS 9.4 software (SAS Institute Inc., Cary, NC). The significance level was fixed at 5%.

**Table 1:** Treatment administration in consecutive order.

Patient *i*	1	2	3	4	5	6	7	8	9	10	11	12	13	14	15	16	17
Treatment	C	C	P	P	P	C	C	C	P	P	P	C	C	C	P	C	C
*N*_*C*_(*i* − 1)	0	1	2	2	2	2	3	4	5	5	5	5	6	7	8	8	9
*N*_*P*_(*i* − 1)	0	0	0	1	2	3	3	3	3	4	5	6	6	6	6	7	7
*τ*_*i*,*TTE*_	1	*δ*	*δ*	*δ*	1	1δ	1	*δ*	*δ*	*δ*	1	1δ	1	*δ*	*δ*	*δ*	*δ*
*τ*_*i*,*CONT*_	0	−*η*	−*η*	−*η*	0	*η*	0	−*η*	−*η*	−*η*	0	*η*	0	−*η*	−*η*	−*η*	−*η*
18	19	20	21	22	23	24	25	26	27	28	29	30	31	32	33	34	35
C	P	P	C	P	P	P	P	C	C	P	P	P	P	C	C	P	C
10	11	11	11	12	12	12	12	12	13	14	14	14	14	14	15	16	16
7	7	8	9	9	10	11	12	13	13	13	14	15	16	17	17	17	18
*δ*	*δ*	*δ*	*δ*	*δ*	*δ*	*δ*	1	1δ	1	*δ*	1	1δ	1δ	1δ	1δ	1δ	1δ
−*η*	−*η*	−*η*	−*η*	−*η*	−*η*	−*η*	0	*η*	0	−*η*	0	*η*	*η*	*η*	*η*	*η*	*η*

For treatment administration in consecutive order 35 patients (18 preventive, 17 conventional) are being regarded from the randomized part of EPISTOP trial.

#### Time to first seizure

As the primary endpoint time to first seizure is a time-to-event outcome a cox proportional hazards model (PROC PHREG in SAS) is used to perform the reanalysis. Time from birth to first seizure is considered as dependent variable. Treatment group and study site are included as predictors (model Ia). Subsequently, the model is extended for bias correction by including the bias effect *τ*_*i*,*TTE*_ as an additional categorical predictor (model Ib). For this purpose we use the biasing policy presented by Rückbeil et al [22] for the statistical analysis. Thus, for the *i*-th patient, the bias effect is represented by

$$\begin{array}{c} \tau_{i,TTE}=\{\begin{array}{cc} \delta & \text{,}\;\text{if}\;N_{P}\left(i-1\right)<N_{C}\left(i-1\right)\\ 1 & \text{,}\;\text{if}\;N_{P}\left(i-1\right)=N_{C}\left(i-1\right)\\ 1\delta & \text{,}\;\text{if}\;N_{P}\left(i-1\right)\\ N_{C}\left(i-1\right), \end{array} \end{array}$$

where *δ* ∈ (0, 1) is the biasing factor. Through the objective of survival prolonging of treatments, patients are identified by *τ*_*i*,*TTE*_ as having either good (τi,TTE=1δ), neutral (*τ*_*i*,*TTE*_ = 1), or poor (*τ*_*i*,*TTE*_ = *δ*) expected response. When *δ* approaches 0, it indicates that patients with poor expected response will experience an event in a short time, while patients with good expected response are not likely to experience an event. On the other hand, if *δ* is close to 1, this reflects the fact that all patients are approximately comparable in terms of health status.

In a sensitivity analysis, the COX regression model is modified for the dependent variable time from study entry to first seizure. The predictors treatment group, study site (model IIa), and bias correction by inclusion of *τ*_*i*,*TTE*_ (model IIb) are modeled analogously as before. For description, the numbers and proportions of patients who had a seizure are given, as well as the median time to seizure in days. Kaplan Meier curves (model Ia and model IIa) are presented for graphical illustration (PROC LIFETEST in SAS). To evaluate the treatment effect, the respective hazard ratios of conservative versus preventive treatment, as well as the corresponding 95%-confidence intervals and p-values, are reported in all models mentioned.

#### BSID

As the secondary endpoints measuring the neurodevelopmental delay at the age of two year assessed using the continuous BSID-III cognitive score and its separate components an ANOVA model (PROC GLM in SAS) is used to perform the reanalysis. The BSID-III cognitive score or one of the separate components at two years of age is considered as dependent variable. Treatment group and study site are included as predictors (model IIIa). Subsequently, the model is extended for bias correction by including the bias effect *τ*_*i*,*CONT*_ as an additional categorical predictor (model IIIb). For this purpose, we use the biasing policy presented by Hilgers et al [23] for the statistical analysis. Thus, for the *i*-th patient, the bias effect is represented by

$$\begin{array}{c} \tau_{i,CONT}=\{\begin{array}{cc} -\eta & \text{,}\;\text{if}\;N_{P}\left(i-1\right)<N_{C}\left(i-1\right)\\ 0 & \text{,}\;\text{if}\;N_{P}\left(i-1\right)=N_{C}\left(i-1\right)\\ \eta & \text{,}\;\text{if}\;N_{P}\left(i-1\right)>N_{C}\left(i-1\right), \end{array} \end{array}$$

where *η* is the biasing factor and represents amount of increase or decrease in the expected response of the *i*-th patient. For description, mean and standard deviation of the BSID-III cognitive score at two years of age for each treatment group are displayed. To evaluate the treatment effect, the respective estimated mean difference between conservative and preventive goups, as well as the corresponding 95%-confidence intervals and p-values, are reported in all models mentioned.

## Results

### Primary study endpoint

**Fig 1:**
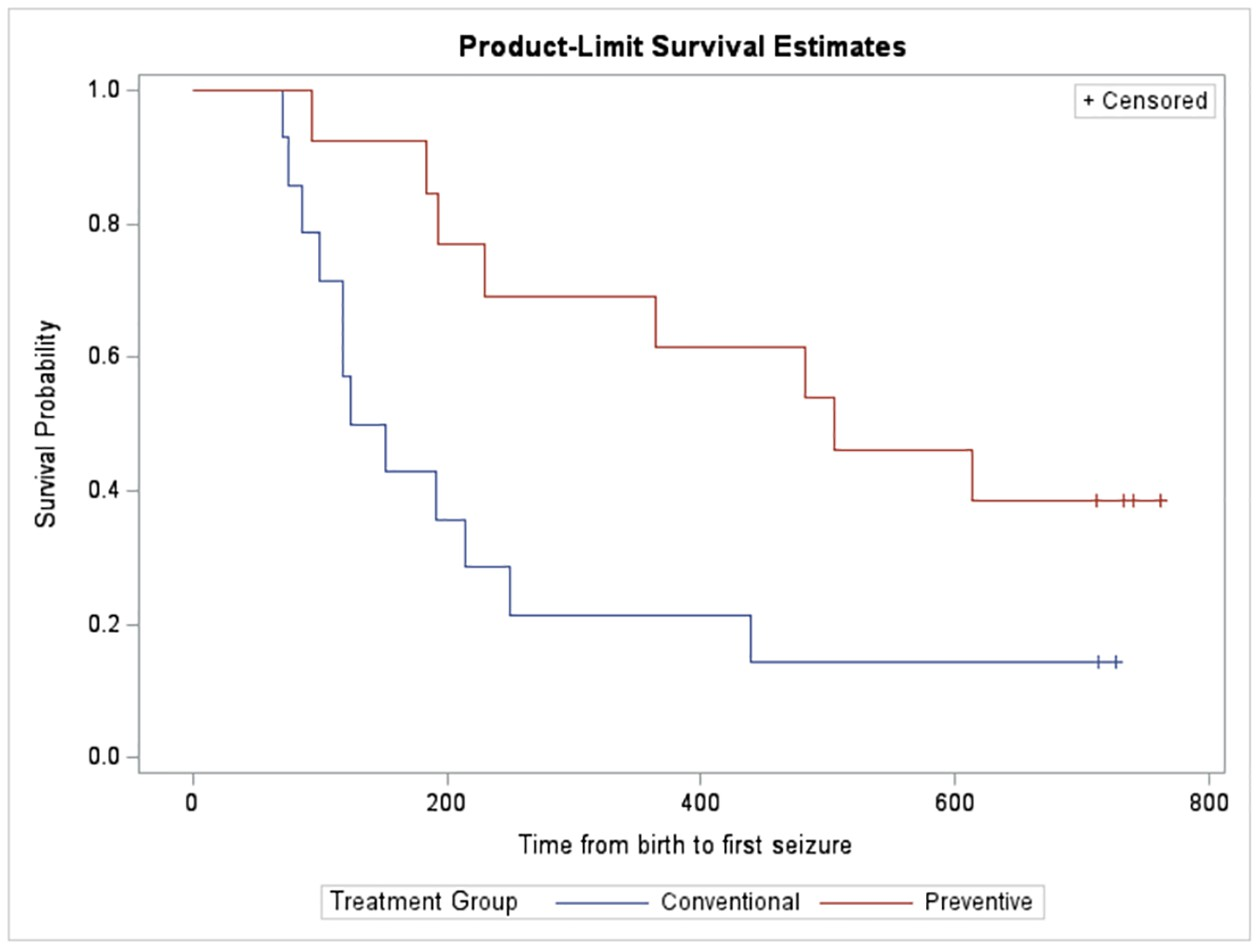
Kaplan-Meier curves for time from birth to first seizures. Time (in days) from birth to first clinical epileptic seizures in EPISTOP patients receiving preventive or conventional vigabatrin.

**Fig 2:**
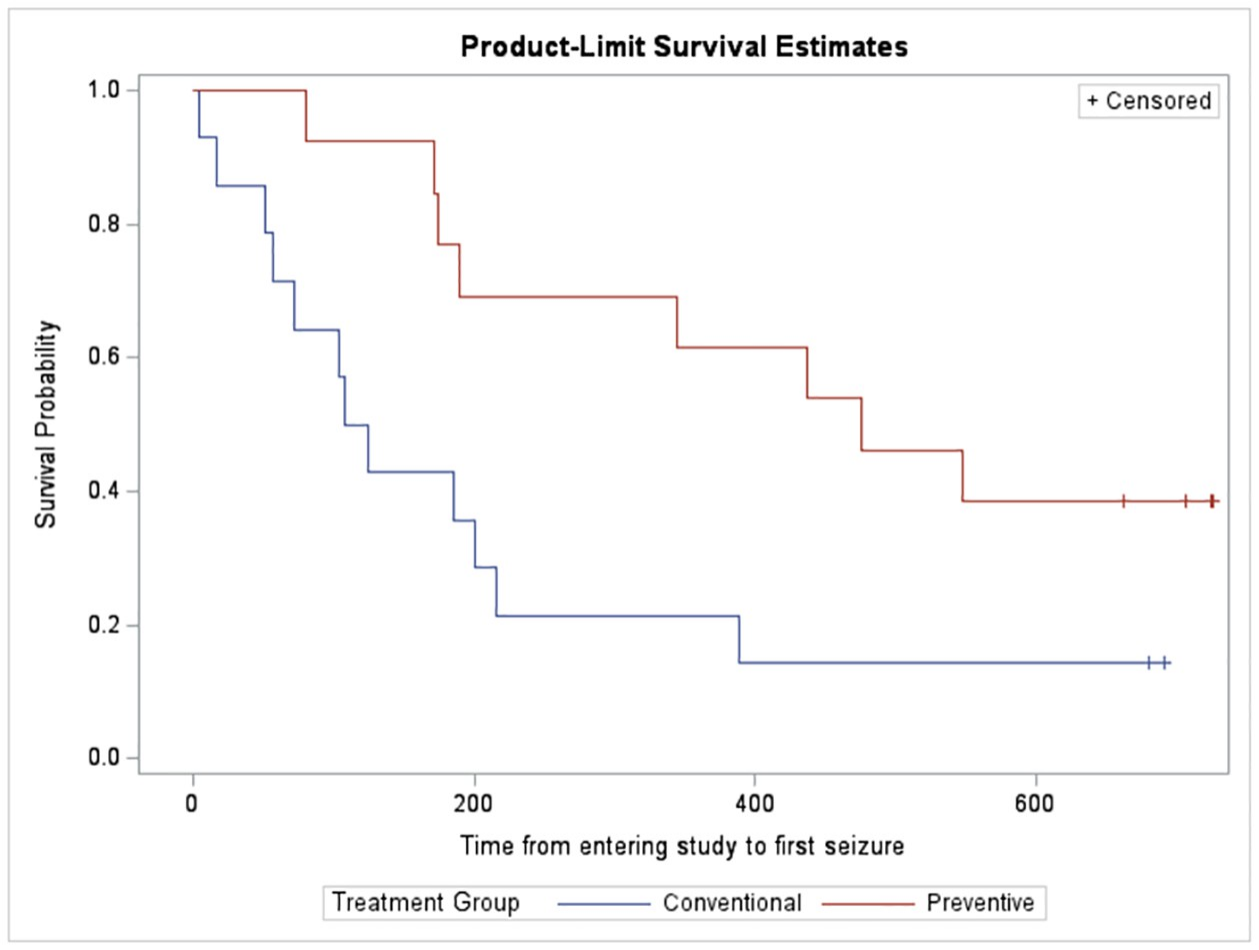
Kaplan-Meier curves for time from entering study to first seizures. Time (in days) from entering study to first clinical epileptic seizures in EPISTOP patients receiving preventive or conventional vigabatrin.

Of the 27 patients included in the randomized part of the trial, 13 were randomly assigned to preventive treatment and 14 to conventional treatment with vigabatrin. In the preventive group, 8 patients experienced at least one seizure and in the conventional group, 12. The median time from birth or study entry to first seizure is is considerably increased under preventive administration of vigabatrin which appears to be associated with a prolonging effect of time (see Table 2). This is also graphically illustrated by the Kaplan-Meier curves from Figs 1 and 2, since for both cases the survival curves between the treatment groups do not overlap.

**Table 2:** Description of primary enpoint.

Treatment	Seizure, n (%)	median time to seizure in days	
		from birth	from study entry
preventive (N = 13)	8 (62)	505.0	476.0
conventional (N = 14)	12 (86)	137.5	116.5

Description of time from birth and time from study entry to first seizure and number of patients with seizures.

Based on the results in Table 3, the previous study results can be reproduced. The uncorrected models Ia and IIa show hazard ratios of 2.91 (95%-CI from 1.11 to 7.67, p-value 0.0306) and 2.89 (95%-CI from 1.10 to 7.58, p-value 0.0316), respectively. It can be concluded that the treatment effect demonstrates a statistically significant benefit of preventive treatment with vigabatrin, regardless of whether the time is considered from birth or study entry to first seizure. A look at the bias-corrected analyses (models Ib and IIb) shows that the estimated hazard ratio and the associated confidence intervals are in very similar ranges (2.82 for model Ib and 2.77 for model IIb).

**Table 3:** Results of primary endpoint analysis.

Model	HR	95%-CI	p-value
Ia	2.91	1.11 to 7.67	0.0306
Ib	2.82	1.05 to 7.53	0.0390
IIa	2.89	1.10 to 7.58	0.0316
IIb	2.77	1.04 to 7.40	0.0425

Comparison between model Ia: published main analysis for time from birth to first seizure, model Ib: bias corrected model for time from birth to first seizure, model IIa: sensitivity analysis for time from entering study to first seizure and model IIb: bias corrected sensitivity analysis for time from entering study to first seizure.

### Secondary study endpoints

In the preventive group the mean BSID-III congnitive score at 2 years of age was 72.92 and 73.33 in the conventional group (see Table 4). Means and standard deviations of the individual components of the BSID-III congnitive score at 2 years of age are also shown in Table 4 separately for each treatment group. In Table 5, the results of the reanalysis (model IIIa) and of the bias corrected analysis (model IIIb) are displayed. For the BSID-III congnitive score at 2 years of age the uncorrected model IIIa shows an estimated mean difference of 4.90 (95%-CI from -5.58 to 15.38, p-value 0.3401). It can be concluded that the treatment effect demonstrates no statistically significant benefit of the preventive treatment with vigabatrin compared to the conventional treatment. A look at the bias-corrected analyses model IIIb confirms this result, as the estimated mean difference of 5.01 and the associated 95%-confidence interval (from -6.04 to 16.06) are in very similar ranges and reflect again a non-statistically significant benefit of preventive versus conventional treatment. The results of the individual components of the BSID-III cognitive score at 2 years of age displayed in Table 5 support this tendency. For the components BSID Motor, BSID Fine Motor, BSID Gross Motor, BSID Language, BSID R. Language, BSID E. Language and BSID GAC we found no statistically significant benefits of the preventive treatment with vigabatrin compared to conventional treatment can be found, as the corresponding 95%-confidence intervals cover the no effect value 0, which is also reflected by the corresponding p-values > 0.05. The BSID SE value alone indicates a significant advantage of preventive over conventional therapy with vigabatrin. However this result is observed in both models IIIa and IIIb, as both 95%-confidence intervals (model IIIa: from 1.51 to 46.67, model IIIb from 5.37 to 48.40) do not cover the no effect value of 0. Again this is well reflected by the corresponding p-values < 0.05 (model IIIa: 0.0378, model IIIb: 0.0175).

**Table 4:** Description of BSID secondary endpoints.

	Treatment	
	preventive	conventional
BSID Cognitive	72.92 (16.16)	73.33 (15.28)
BSID Motor	72.92 (16.28)	70.5 (13.94)
BSID Fine Motor	5.25 (2.77)	5.33 (3.34)
BSID Gross Motor	4.92 (2.11)	5.92 (2.27)
BSID Language	64.42 (12.57)	70.67 (15.84)
BSID R. Language	4.5 (3.06)	5.00 (2.80)
BSID E. Language	3.33 (1.61)	4.67 (2.57)
BSID SE	78.78 (15.37)	100.00 (28.84)
BSID GAC	65.33 (18.56)	76.50 (23.05)

Mean and SD of BSID secondary endpoints at two years of age in treatment groups.

**Table 5:** Results of secondary endpoint analysis.

Secondary Endpoint	Model	MD	95%-CI	p-value
BSID Cognitive	IIIa	4.90	-5.58 to 15.38	0.3401
	IIIb	5.01	-6.04 to 16.06	0.3520
BBSID Motor	IIIa	4.85	-7.11 to 16.81	0.4067
	IIIb	4.28	-8.32 to 16.88	0.4835
BSID Fine Motor	IIIa	0.62	-1.58 to 2.81	0.5640
	IIIb	0.55	-1.78 to 2.88	0.6246
BSID Gross Motor	IIIa	1.30	-0.66 to 3.26	0.1803
	IIIb	1.22	-0.86 to 3.29	0.2330
BSID Language	IIIa	6.64	-0.99 to 14.26	0.0844
	IIIb	6.92	-1.18 to 15.03	0.0894
BSID R. Language	IIIa	1.16	-0.08 to 2.40	0.0650
	IIIb	1.18	-0.15 to 2.51	0.0775
BSID E. Language	IIIa	0.78	-0.48 to 2.04	0.2117
	IIIb	0.82	-0.52 to 2.16	0.2154
BSID SE	IIIa	24.09	1.51 to 46.67	0.0378
	IIIb	26.88	5.37 to 48.40	0.0175
BSID GAC	IIIa	7.23	-12.97 to 27.43	0.4629
	IIIb	0.82	-0.52 to 2.16	0.2154

Comparison between Model IIIa: main analysis for BSID secondary endpoint at two years of age and Model IIIb: Bias corrected analysis. MD: estimated mean difference of conserverative—preventive group.

## Discussion

Several factors in clinical trials can result in biased study findings. Along with selection bias, which is the main focus of this paper, other biases can also occur. In clinical trials for rare diseases with no improvement, it’s problematic that blinded study personnel can observe when patients don’t progress or improve. This unintentional unblinding can lead to patients being treated differently based on their assigned treatment. Additionally, the trial sponsor may decide that patients aren’t improving and stop for futility or even change the eligibility criteria. EPISTOP trial was an innovative trial proposing a classical RCT to validate observations of improved outcomes in patients with TSC complex treated preventively with ASM before experiencing seizures. Additionally, the study sought to gain insights into the biomarkers related to seizures and cognitive decline in those patients [21]. To overcome the rarity of this condition, a multicenter study was conducted within an EU network and it was intended to perform the randomized controlled trial (RCT) at 10 enrolment sites. However, two major factors dictated the number and origin of patients included in each group (RCT or OLT) in this study: the different national scientific visions on clinical trial design and the different national ethical rules and laws in regard to to preventive therapies and to TOPFA practice (termination of pregnancy for fetal anomaly) [24] that is applied to prenatal diagnosis of TSC in some countries [25, 26].

### The impact of the national cultural vision on clinical trial design (4 sites didn’t consider the RCT design as ethic)

Diverse national cultural perspectives regarding trial design resulted in different viewpoints on the ethical conduct of this study in each country. Cultural, ethical, legal, and regulatory disparities specific to each country shaped the understanding of ethical considerations and influenced the acceptance or rejection of the RCT’s design as ethically appropriate. Thus, the RCT was not approved by ethics boards at 4 sites: in 2 the benefit of the preventive treatment was accepted and there were no further needs for a trial and decreasing the chances for the patients under placebo arm. For others preventive treatment was not accepted and ASM should be used after seizures occurrence to insure a risk/benefit balance from the treatment. Hence subjects at those sites were enrolled in a parallel open-label trial (OLT), with treatment according to local clinical practice: preventive treatment at 2 sites and conventional treatment at 2 others. The study finally ended including 27 patients in the randomized trial and 27 patients in the open-label trial. The intercountry ethical discrepancy regarding trial design highlights the importance of addressing cross-cultural research issues that pushed some stakeholders in the field to suggest developing an ethical framework to assist policymakers in navigating crucial ethical concerns [27]. Additionally, this regional ethical disparity in Europe encouraged the European Clinical Research Infrastructure Network (ECRIN) to centralize resources by establishing and updating the Regulatory and Ethical Tool (CAMPUS), a central resource for information about clinical trial regulatory and ethical requirements covering 22 European countries and multiple study types [28].

### The impact of the national cultural vision on the population of includible individuals (prenatal diagnoses of TSC lead to different attitudes according to societal views about medical termination of pregnancy)

Although medical and technical advancements resulted in increased prenatal diagnosis and detection rates of a wide range of abnormalities [29, 30], limited therapeutic options are yet to be offered, leading to difficult situations of choosing between the continuation of pregnancy and abortion. But such decisions depend heavily on the laws and practices that differ locally [31] and in the 113 (57%) countries around the world where it is legal [32, 33]. These variable attitudes across countries regarding the permissibility of termination of pregnancy are based on cultural, religious, and ethical beliefs that play a substantial role in shaping public opinion and policy surrounding prenatal care and abortion. This will affect the representations of different ethnic groups from different countries impeding for instance the generalizability of the results to all ethnic and racial groups.

### Reuse of data to reinforce the result of CT

This study capitalized on data reuse, a major priority in the area of rare diseases as performing RCTs for each rare or ultrarare disease is simply impossible [34]. The statistical re-analysis of this trial raw data supports the published results and reinforces that there is no additional bias introduced by randomization, thereby increasing the value and the generalisability of the results. This reanalysis on the bias of the randomization did not show any pitfalls but sheds light on the need of the consideration of this aspect in further trial designs, mainly in small samples in order to avoid any additional bias in already a small number sample where significance might be difficult to achieve. This emphasizes the urgent need for early collaboration between methodologists with expertise in small samples and clinicians or industry intending to develop clinical trials in RDs. In addition to reanalyzing the data as described, there are other methods available for quantifying selection bias. For instance, the Berger Exner test [35] can be used to detect third-order selection bias. Furthermore, time-to-event outcomes can be reanalyzed using design based inference approaches, such as the asymptotic permutation-based logrank tests outlined in [36]. The utilization of their R-package “interval” resulted in a p-value of 0.02934, which supports the findings presented in Table 3. In particular, design based inference, as a distribution-free method, appears to be a promising approach, especially in situations with small sample sizes where the assumption of a population model or specific distribution requirements does not seem justified.

### Innovation and acceleration in the development of drugs in rare diseases

Approximately 26 million individuals in the EU (1 in 17 people) suffer from rare diseases, causing a substantial unmet medical need and a serious public health challenge [37]. Additionally, only 5% or fewer of the 6000–8000 rare diseases are estimated to have at least one approved treatment—known as “orphan” therapies. This is due to the fact that bringing RD treatments to the market is a lengthy process, hampered by factors such as limited knowledge, regulatory obstacles, safety concerns, financial risks associated with developing trials for small populations, and a dearth of systematic implementation of best practices. This delay leaves many serious and life-threatening diseases lacking meaningful treatment endangering the life of affected patients while awaiting treatment approval. Fortunately, many initiatives were taken to hasten access to treatment in the RD population. Regulatory agencies have devised strategies to help bring innovation to patients, through pathways such as the Accelerated Approval Program [38], and by establishing programs speeding and increasing the development of effective and safe treatment options to address the unmet needs of patients with rare diseases [39]. Furthermore, the EMA offers incentives to encourage companies to research and develop medicines for rare diseases, and to apply for orphan designation for their medicine, with a special committee, the Committee for Orphan Medicinal Products (COMP) being in charge of reviewing applications for orphan designation [40]. EMA launched also a pilot project to support the repurposing of medicines in order to support not-for-profit organizations and academia to gather or generate sufficient evidence on the use of an established medicine in a new indication with the view to have this new use formally authorized by a regulatory authority [41]. Additional initiatives to boost the advancement of rare disease therapies were taken by the IRDiRC through the development of the Orphan Drug Development Guidebook which sought to draw together all the knowledge on developing drugs for rare diseases and build a framework for optimal use of existing tools available in Europe, Japan, and the US—ultimately inspiring a “quantum change” in the way drugs are developed [42]. Furthermore, IRDiRC set up a Small Population Clinical Trials (SPCT) Task Force that came up with numerous recommendations regarding the alternative trial design use in RD CT (rather than classical RCT), the need to combine different sources of safety data, the consideration multi-arm trials in rare diseases therapy development, and the importance of having funders support, patient engagement, and regulatory input throughout clinical development [13]. The EJP RD, through the demonstration and innovation projects, by validating previous innovative statistical methods for small-population clinical trials (IDeAl [14], asterix [15] and InSPiRe [16], and proposing the development of new ones, tackling the urgent needs, actively contributed to the innovation in RD research. The ultimate goal is to get regulatory approval or validation of these innovative statistical methodologies so they can be used to accelerate the drug development phases to achieve faster regulatory approval and patients’ reach leaving no one behind.

## Supporting information

S1 File EPISTOP clinical trial protocol. (PDF)

S2 File CONSORT checklist. (PDF)
