# Supplementary material for: Methodological insights from the EPISTOP trial to designing clinical trials in rare diseases—A secondary analysis of a randomized clinical trial
Source: PLoS One. 2024 Dec 3;19(12):e0312936. doi: 10.1371/journal.pone.0312936 (PMC11614242; doi:10.1371/journal.pone.0312936)
Supplement: S1 File — (PDF) [file pone.0312936.s001.pdf]

<u>Final version</u> of the protocol of the clinical part of EPISTOP project

Long-term, prospective study evaluating clinical and molecular biomarkers of epileptogenesis in a genetic model of epilepsy – tuberous sclerosis complex

Authors: Katarzyna Kotulska, Sergiusz Jóźwiak

Version number : Final 05

Release date: March 2016

Property of EPISTOP consortium. Confidential. May not be used, divulged, published or otherwise disclosed without the consent of EPISTOP consortium

Table of contents

LIST OF ABBREVIATIONS **3**

PROTOCOL SUMMARY **4**

STUDY OBJECTIVES 4
STUDY POPULATION 4
OVERVIEW OF STUDY DESIGN 4
STATISTICAL CONSIDERATIONS 5

BACKGROUND **6**

TUBEROUS SCLEROSIS COMPLEX AS A GENETIC MODEL OF EPILEPSY 8
AN OPPORTUNITY TO CONDUCT PROSPECTIVE STUDIES OF THE LATENT PHASE OF EPILEPTOGENESIS IN TSC PATIENTS 8
EPILEPTOGENESIS BEYOND THE LATENT PHASE: MODEL OF DRUG-RESISTANCE EPILEPSY 9

RATIONALE **9**

RATIONALE FOR THE STUDY DESIGN 9
RATIONALE FOR THE TREATMENT OF ELECTROENCEPHALOGRAPHIC EPILEPTIFORM DISCHARGES 11

OBJECTIVES AND ENDPOINTS **12**

CLINICAL STUDY DESIGN **12**

INCLUSION CRITERIA 12
EXCLUSION CRITERIA 13
STUDY OVERVIEW 13
STUDY PROCEDURES 16
Video EEG *16*
MRI *17*
Neuropsychological assessment *18*
Blood biomarkers collection *19*
VISIT SCHEDULE 19
Baseline Visit *19*
Next visits *20*
Follow-up visit *20*
PATIENTS' NUMBERING 21
RANDOMIZATION PROCEDURES 21

ADVERSE EVENTS **21**

DATA COLLECTION AND MANAGEMENT **22**

MONITORING PLAN **23**

DATA QUALITY ASSURANCE **24**

EARLY STOPPING RULES **25**

WITHDRAWAL OF INDIVIDUAL SUBJECTS **25**

STATISTICAL ANALYSES **25**

ETHICAL CONSIDERATIONS **26**

INFORMED CONSENT PROCEDURES **27**

PERSONAL DATA PROTECTION **27**

REFERENCES **30**

APPENDICES **32**

List of Abbreviations

AE – Adverse Event

AED – AntiEpileptic Drug

ANCOVA – Analysis of Covariance

CI – Confidential Interval

CRO – Contract Research Organization

CRF – Case Report Form

DNA – Deoxyribonucleic Acid

EEG – Electroencephalogram

IPCZD – The Children's Memorial Health Institute, Warsaw, Poland

mRNA – messenger Ribonucleic Acid

mTOR – mammalian Target of Rapamycin

RCT – Randomized Clinical Trial

RNA – Ribonucleic Acid

SS – Subclinical Seizure

TSC - Tuberous Sclerosis Complex

vEEG – video Electroencephalography

Protocol Summary

Study title: Long-term, prospective study evaluating clinical and molecular biomarkers of epileptogenesis in a genetic model of epilepsy – tuberous sclerosis complex.

Study objectives

The primary objective of clinical part of EPISTOP project is to identify the clinical and molecular biomarkers of epileptogenesis in a prospective clinical study of patients with TSC.

Secondary objective of the clinical part of EPISTOP is to compare the neurodevelopmental outcome in patients diagnosed as having epilepsy after clinical or subclinical seizures vs after electroencephalographic epileptiform discharges but prior to clinical or subclinical seizures, in a randomized trial in TSC patients.

Study population

The target population is comprised of about 100 TSC male or female infants with a definite diagnosis of TSC, aged up to 4 months, with no clinical seizures seen by caregivers or clinical or subclinical seizures on videoEEG recording on baseline. Forty age-matched non-TSC infants will be enrolled to a control group.

Overview of study design

This is a prospective study of epileptogenesis in TSC infants. In control subjects only one blood sampling will be performed and those infants will not be observed prospectively.

The study consists of two phases: (1) prospective tracking of epileptogenesis by means of serial vEEG recordings; (2) treatment of epilepsy diagnosed after clinical or after electroencephalographic epileptiform discharges but before seizures.

All patients enrolled in the study will participate in the first phase. The second phase will concern only the children with epilepsy with electroencephalographic epileptiform discharges and/or clinical or subclinical seizures, whose parents/caregivers will give consent for the randomized part of the project.

At baseline, all patients will undergo neuroimaging examination by means of MRI, a battery of neuropsychological tests, blood biomarker sampling, and the review of medical history of the patient and the family.

Epileptogenesis in TSC infants will be tracked by means of serial vEEG recordings. In children with diagnosed epilepsy, standard therapy with recommended first line antiepileptic drug will be given. Children with clinical seizures, either noticed by a caregiver, or a treating neurologist or clinical or subclinical seizures recorded on video during vEEG will be immediately diagnosed as having epilepsy. Infants that have epileptiform discharges on vEEG

and no clinical or subclinical seizures, if their parents/caregivers give consent, will enter the randomized part of the study. Those children will be randomized into two groups: group A will be diagnosed as having epilepsy after electroencephalographic epileptiform discharges but before subclinical or clinical seizures, and the patients in group B will be diagnosed as epileptic after clinical or subclinical seizures appear. Epileptiform discharges on EEG will be graded according to a EPISTOP scale (Appendix 3), using EPISTOP software.

All infants diagnosed with epilepsy will receive standard therapy with recommended first line antiepileptic drug starting from the day of diagnosis.

Children whose parents/caregivers will not give consent for the randomized part of the project, will be followed with serial vEEG and epilepsy will be diagnosed after clinical or subclinical seizures.

Children without seizures and no epileptiform discharges on vEEG will be followed without treatment.

Blood samples for biomarker studies will be collected at study entry, at the onset of epileptiform discharges on vEEG or at the age of 6 months, whichever is applicable, at the onset of clinical/subclinical seizures, and at the end of follow-up (age 2 years) in all patients participating in the project.

At the age of 24 months, all TSC infants participating in the study will undergo neuroimaging examination by means of MRI, a battery of neuropsychological tests, and epilepsy analysis.

Primary Endpoint of the study is the collection of a set of molecular and clinical biomarkers in full analysis set of patients.

Key secondary endpoint is better neurodevelopmental outcome in patients diagnosed as epileptic after electroencephalographic epileptiform discharges in comparison to patients diagnosed as having epilepsy after the onset of seizures. The parameters of neurodevelopmental outcome include: the risk of seizures, the distribution of seizure free patients at the age of 24 months, proportion of patients with drug resistant seizures at the age of 24 months, proportion of patients with normalized EEG at the age of 24 months, time to seizures onset, and the neuropsychological outcome recognized as the results in a battery of tests performed at the age of 24 months.

Statistical considerations

Full analysis set comprises all patients participating in the study, including the control group. This set will be divided into subsets: control group, TSC patients with epilepsy, and TSC patients with no epilepsy. Among TSC patients with epilepsy, patients with well-controlled

seizures and patients with drug-resistant epilepsy will be identified. In full analysis set the blood biomarkers will be analysed. Clinical analysis set will comprise of all TSC infants enrolled in the study and the clinical biomarkers of epileptogenesis (neuroimaging, vEEG, data from medical history) will be analysed in this set. Outcome analysis set will comprise of infants participating in the randomized part of the study and the neurodevelopmental outcome in respect to the point of epilepsy diagnosis (electroencephalographic epileptiform discharges onset in group A and seizures onset in group B) will be assessed in this set.

The interim analyses will be performed when 70% of the patients will complete the whole study. Final analyses will be performed when the last patient will complete the study (at the age of 24 months).

Background

Epilepsy affects 1% of the world's population. In Europe, 6 million people have epilepsy (*World Health Organization, 2010*). The estimated total cost of €13.8 billion in European Union in 2010 makes epilepsy a significant socioeconomic burden at individual, family, health services, and society level (*Olesen, 2012*). Despite great progress in the management of epilepsy and increasing numbers of antiepileptic drugs, 30-40% of epilepsy patients are refractory to all available medications and many suffer from epilepsy-related comorbidities (*Jensen, 2011*). This group of patients accounts for 80% of the cost of epilepsy management (*Begley, 2000*).

In more than 65% of patients, epilepsy begins in childhood and the incidence of epilepsy is highest in the first year of life (*Hauser, 1993*). In children, the problem of epilepsy is far beyond seizures, as about 50% of children with epilepsy suffer from psychiatric and behavioural comorbidities, including developmental delay, learning disabilities, and autism spectrum disorder (*Ono, 2012*).

Furthermore, early onset of seizures is regarded as one of the major risk factors for development of drug-resistant epilepsy. The risk of severe neurodevelopmental delay and antiepileptic drug (AED)-resistance is particularly high in children with infantile spasms, which account for about 25% of early childhood epilepsies.

Major limitation for all current antiepileptic drugs is that they act primarily on the molecular mechanisms that are already established in the patient when epilepsy is diagnosed. Thus far, no medication has resulted in the significant reduction of the number of patients with drug-resistant epilepsy.

According to the existing definition, epilepsy requires the occurrence of at least two epileptic

seizures (*Fisher, 2005*). However, it is now widely accepted that clinical seizures are preceded by a latent period of epileptogenesis (*Pitkanen, 2011*, *Rakhade, 2009*). This cascade of cellular and molecular events may be triggered by diverse brain insults, including trauma, infection or genetic predisposition, and leads to the formation of hyperexcitable neural networks ultimately resulting in spontaneous epileptiform activity. This process continues with onset of clinical seizures, leading to the development of established, drug-resistant epilepsy, and secondary comorbidities (Fig. 1). Thus far, studies aimed at understanding the molecular and cellular mechanisms of epileptogenesis have been possible only in animal models. In humans, epileptogenesis studies are difficult because the patients usually present **after** seizures and little is known about the earlier stages of the disease. The molecular changes occurring during epileptogenesis in animal models are still not fully understood, but are known to include changes in gene expression, activation of several immune and inflammatory processes, and others. Although animal models are useful, there is an urgent need for better understanding of epileptogenesis in humans.

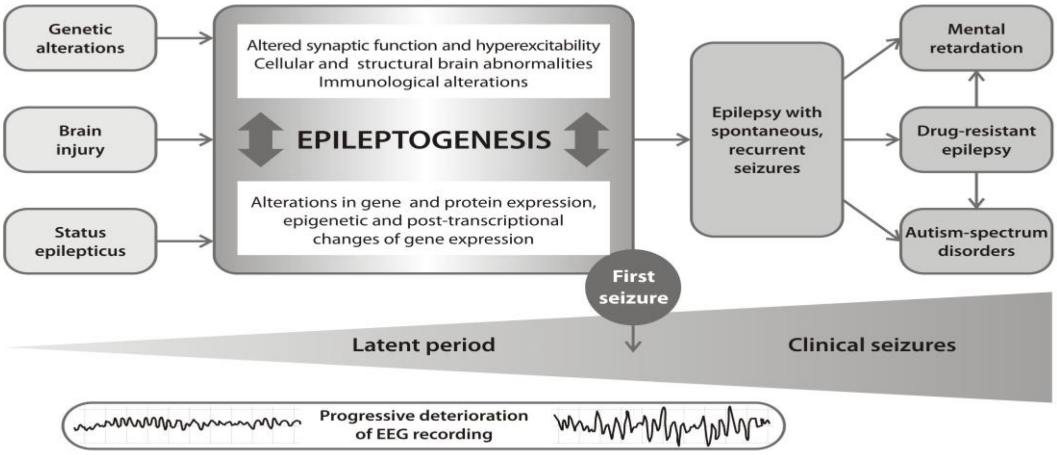

Fig.1. Epileptogenesis progress. Brain insult (e.g., injury, genetic predisposition, experimental status epilepticus) induces a cascade of cellular and molecular events leading to the clinical manifestation of seizures. This process can be tracked on EEG. Epileptogenesis extends beyond the first seizure and contributes to the development of neuropsychiatric comorbidities of epilepsy.

Currently, there are no recognized biomarkers enabling the diagnosis of epilepsy before the clinical seizure appear. There is also no established antiepileptogenic strategy or medication to prevent the development of epilepsy and its co-morbidities. Introduction of novel antiepileptogenic drugs is not possible due to the poor understanding of epileptogenesis itself and a shortage of specific targets for such drugs. Attempts to use standard antiepileptic drugs in a preventative manner in most cases have not been successful (*Mani, 2011*). Lessons from

posttraumatic epilepsy studies show, that even in patients with a well-defined risk factor for epilepsy like head trauma, it is extremely difficult to predict the onset of seizures. Posttraumatic epilepsy is recognized as the major cause of seizures in adults. The risk of epilepsy in patients after head injury varies from 4 to 53% and depends on many, not yet fully understood, factors (*Christensen, 2012*). Recent data show that the risk of epilepsy remains increased even after 10 years following the triggering trauma (*Christensen, 2009*). Considering these data, preventative treatment trials for post-traumatic epilepsy would include many patients who are at low risk for a long period of time, thus making the risk/benefit ratio unfavourable. Therefore, there is an urgent need for identification of clinically applicable biomarker risk factors for epilepsy development. Such biomarkers should be available from blood samples or non-invasive clinical procedures, like neuroimaging or EEG examinations.

Tuberous sclerosis complex as a genetic model of epilepsy

Tuberous Sclerosis Complex (TSC) is a genetically determined neurocutaneous syndrome affecting 1 child in 6,000 *(Curatolo, 2008).* Molecular genetic studies have shown that there are two genes that cause TSC, *TSC1* and *TSC2*, both of which are subject to heterozygous inactivating mutations in TSC. The hallmark of the disease is the occurrence of cortical tubers and subependymal nodules. Cells in TSC lesions show constitutive hyperactivation of mTOR due to loss of TSC1/TSC2 inhibition of mTOR through rheb. TSC is often considered an excellent clinical model of severe focal epilepsy, as 70 to 90% of patients are affected by epilepsy and in most cases the seizures are drug-resistant. In the majority of patients epilepsy manifests in the first months of life and half of patients develop cognitive impairment, autism spectrum disorder or other neurodevelopmental disturbances *(Jozwiak, 1998).* Epilepsy in TSC is often focal initially, but in many cases infantile spasms follow or coexist with focal seizures. Thirty-eight percent of TSC patients experience infantile spasms, and TSC accounts for 10% of all infantile spasms cases (*Osborne, 2010*). Therefore TSC is an excellent model for both focal epilepsy and infantile spasms. Although there is definite clinical heterogeneity, TSC represents a relatively homogenous group of patients for the studies of epileptogenesis, who are at high risk of this disease.

An opportunity to conduct prospective studies of the latent phase of epileptogenesis in TSC patients

Increasing numbers of TSC patients are diagnosed prenatally or soon after birth, through increasing awareness of this syndrome, and early detection of cardiac rhabdomyomas which are often detected on routine prenatal echocardiography. These tumours are present in over 80% of foetuses with TSC. When cardiac rhabdomyomas are detected, prenatal or neonatal

brain MRI can be performed to confirm the diagnosis of TSC. This early diagnosis enables serial clinical observation before the onset of epilepsy, which usually starts at the age of 4-6 months. A prospective study of TSC infants before clinical seizures showed that 71% of all TSC infants develop epilepsy in the first 24 months of life (*Jozwiak, 2011*).

Epileptogenesis beyond the latent phase: model of drug-resistance epilepsy

The mechanisms of drug-resistance are likely to be multifactorial and are influenced by the age of the patient, the aetiology of epilepsy, and the preceding treatment. Currently, there is limited data on the development of drug-resistance in humans, and the identification of patients at risk is not possible at early stages of epilepsy. In the majority of TSC patients, epilepsy is resistant to standard pharmacotherapy. Published studies indicate that by the age of 24 months, 42% of TSC patients will have drug-resistant seizures (*Chu-Shore, 2010, Jozwiak, 2011).* There is a strong association between mental outcome and epilepsy, both in TSC and in general. The prevalence of neuropsychiatric abnormalities among TSC patients varies from 40 to 70%, and its severe or profound form (defined as IQ lower than 36) is reported in 30-45% of patients. An original comprehensive report on this topic noted that all TSC patients (of 160 studied) with intellectual disability had a history of epilepsy, while none without seizures had mental retardation (*Gomez, 1988*). At the age of 24 months, neurodevelopmental delay is observed in 48% of TSC children, and is seen exclusively in those with seizures (*Jozwiak, 2011*).

Rationale

This study is aimed to identify the clinical and molecular biomarkers of epileptogenesis in humans. The secondary goal of the study is to compare the neurodevelopmental outcome in TSC patients diagnosed as having epilepsy after electroencephalographic epileptiform discharges vs seizures, either clinical or subclinical. The high risk of epilepsy among infants with TSC and the possibility of diagnosis of TSC before the onset of epilepsy justify this model for the prospective study on epileptogenesis.

Rationale for the study design

This study is composed of two phases: (1) prospective tracking of epileptogenesis by means of serial vEEG recordings; (2) management and follow-up of epilepsy diagnosed after seizures or after electroencephalographic epileptiform discharges but prior to clinical or subclinical seizures. All TSC infants enrolled in the study as well as control children will participate in phase 1, whereas only children diagnosed with epilepsy will participate in phase 2. The time point of epilepsy diagnosis (at the onset of electroencephalographic epileptiform

discharges or clinical/subclinical seizures) will be randomly assigned to participating children by central randomizer and will be blinded to the patients' caregivers and treating neurologists.

The identification of the biomarkers of ongoing epileptogenesis, as well as delineation of the point of no return, at which the occurrence of clinical seizures is inevitable, requires the prospective study, starting before the onset of clinical seizures. To achieve the clinical usefulness of potential biomarkers, they should be based on the analysis of standard clinical tests: neuroimaging by means of MRI, EEG, blood samples.

Our study will use the standard clinical tests to identify the biomarkers of epileptogenesis on neuroimaging studies, EEG, and in the blood samples in TSC infants before and after the onset of seizures, to track the changes in measured parameters during ongoing epileptogenesis. We will compare the results obtained in individual patients before the onset of EEG abnormalities, after the onset of electroencephalographic epileptiform discharges, after seizures and at the age of 24 months. In order to identify the risk of epilepsy among TSC patients, we will compare the results obtained in patients who develop epilepsy and those who remain epilepsy free. We will also compare the results obtained in TSC children with age-matched non-epileptic infants.

We also aim to establish the earliest possible point to diagnose epilepsy in TSC infants. It is now widely accepted that the clinical seizures are preceded by the progressing deterioration of EEG (*Philippi, 2008; Fastenau, 2009*). Such deterioration is not seen in TSC infants who do not develop clinical seizures (*Jozwiak, 2011*). EEG is a standard, non-invasive procedure in epileptic children. Therefore, EEG will be used to track epileptogenesis and to set the points for biomarkers sampling. Patients with epileptiform discharges on EEG recordings will enter the blinded, randomized part of the study, aimed to compare the neurodevelopmental outcome in patients with preclinical diagnosis of epilepsy vs patients with diagnosis after seizures appearance. (Fig.2)

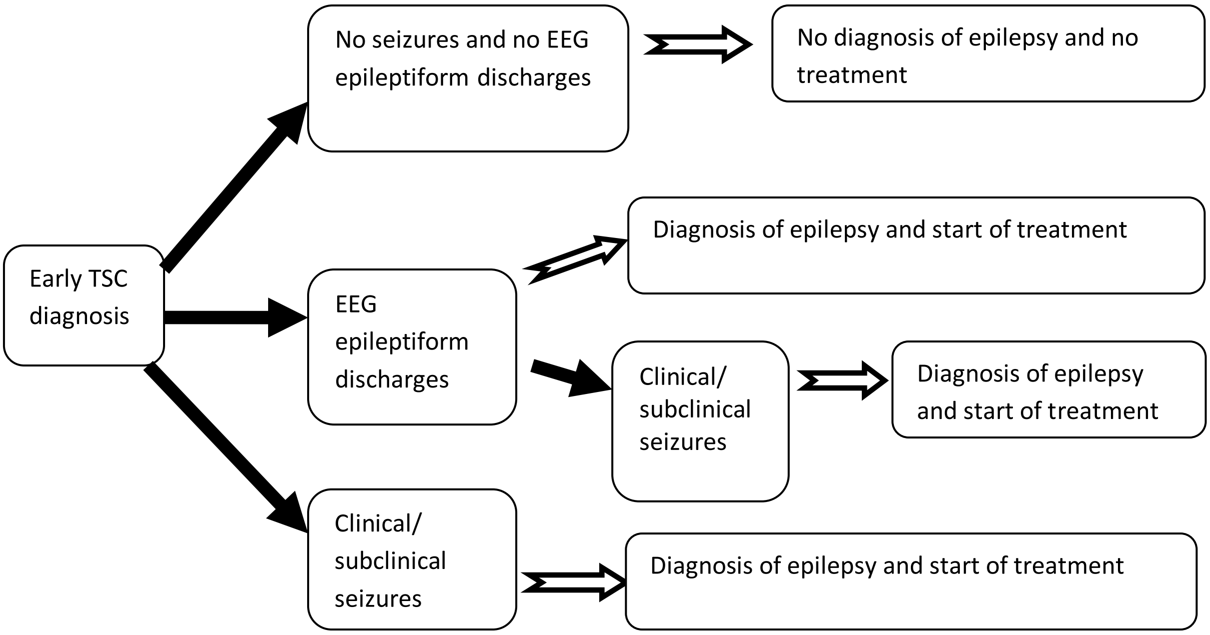

Fig.2 Time points of epilepsy diagnosis and implementation of antiepileptic treatment in TSC infants participating in EPISTOP.

To ascertain blinding of the study, the reports of vEEG recordings will not be sent to treating neurologist, but only to central randomizer (Prof. Lieven Lagae). He will provide the treating neurologist with the diagnosis of epilepsy or no epilepsy, without giving the details of EEG. If the diagnosis of epilepsy is provided, it can mean either of the following: the patient had clinical or subclinical seizures recorded on videoEEG, or had electroencephalographic epileptiform discharges and was randomized to group diagnosed with epilepsy at that point. Similarly, if the treating neurologist receives a diagnosis of no epilepsy in a patient, it is not known whether EEG was normal, or the patient had epileptiform EEG, but was randomized to group diagnosed as epileptic at the onset of clinical seizures.

Taken together, the treating neurologist and the patient's parents/caregivers will be blinded to the diagnostic approach in a patient, but at the same time, an adequate treatment can be implemented.

Rationale for the treatment of electroencephalographic epileptiform discharges

Epileptiform discharges on EEG not only precede clinical seizures, but also reflect changes in the brain that may per se cause neurodevelopmental delay and autism in children (*Primec, 2006*). In animal models it was shown that epileptogenesis could be interfered by antiepileptic treatment implemented before the onset of clinical seizures (*Blumenfeld, 2008*). A recent study (*Jozwiak, 2011*) showed that initiation of antiepileptic treatment before the onset of clinical seizures but after the onset of epileptiform discharges on EEG reduced the risk of

intellectual disability and drug-resistant epilepsy. Amelioration of epilepsy by implementation of antiepileptic drugs before the clinical seizures onset was also shown in neonates with severe hypoxic-ischemic encephalopathy (*van Rooij, 2010*). Current guidelines for epilepsy management in TSC patients recommend treatment of subclinical seizures equally to standard treatment after clinical seizures onset (*Curatolo, 2012*).

Objectives and endpoints

The primary objective of clinical part of EPISTOP project is to identify the clinical and molecular biomarkers of epileptogenesis in a prospective clinical study of patients with TSC.

Secondary objective of the clinical part of EPISTOP is to compare the neurodevelopmental outcome in patients diagnosed as having epilepsy after seizures vs after electroencephalographic epileptiform discharges in a randomized trial in TSC patients.

Primary endpoint of the study is the collection of a set of molecular and clinical biomarkers in full analysis set of patients, including all TSC infants participating in the study and the control subjects. **Key secondary endpoint** is better epilepsy outcome in patients diagnosed as epileptic after electroencephalographic epileptiform discharges in comparison to patients diagnosed as having epilepsy after the onset of seizures, either clinical or subclinical. The parameters of neurodevelopmental outcome include: time to seizures onset, the risk of seizures, the distribution of seizure free patients at the age of 24 months, proportion of patients with drug resistant seizures at the age of 24 months, the risk of infantile spasms, proportion of patients with normalized EEG at the age of 24 months, the neuropsychological outcome recognized as the results in a battery of tests performed at the age of 24 months.

Clinical study design

EPISTOP is a multicenter, prospective study of epileptogenesis in TSC infants. Each patient will participate in the study till the age of 24 months.

Patient population: the target population is comprised of about 100 TSC male or female infants with a definite diagnosis of TSC, aged up to 4 months, with no clinical seizures seen by caregivers or clinical or subclinical seizures on videoEEG recording at baseline. Forty age-matched non-TSC infants will be enrolled to a control group.

Inclusion criteria

Inclusion criteria for TSC patients:

- male or female infants with a definite diagnosis of TSC (Roach criteria; *Roach 1998* or

DNA confirmed),

- age up to 4 months at the moment of enrolment,
- no clinical seizures seen by caregivers
- no seizures (clinical or subclinical) on baseline videoEEG recording,
- written informed consent of caregivers. It is possible to give consent for the observational part of the study only. In this case, the child will not enter the randomized part of the study.

Inclusion criteria for the control group:

- male or female infants who have undergone routine MRI for reasons other than epilepsy and brain tumor or cortical defects,
- age up to 24 months at the moment of study entry,
- written informed consent of caregivers.

Exclusion criteria

Exclusion criteria for TSC patients:

- any type of seizures observed till baseline visit,
- antiepileptic treatment at or prior to study entry,
- contraindications to MRI,
- any severe and/or uncontrolled medical condition that is considered by the investigator as possibly affecting the EPISTOP analyses or procedures.

Exclusion criteria for the control group:

- any sign or symptom suggesting TSC diagnosis,
- any type of seizures observed at study entry,
- antiepileptic treatment at study entry,
- history of seizures, with the exception of febrile seizures,
- any severe and/or uncontrolled medical condition that is considered by the investigator as possibly affecting the EPISTOP analyses or procedures.

Study overview

Patients enrolled in the control group will not be followed in a prospective manner. Only blood biomarkers will be taken from them once in a study. Epileptogenesis will be tracked in all TSC infants participating in the study. The children whose parents/caregivers give consent for the observational part of the project only, will be followed with serial vEEG but the results of the vEEG recordings will not be blinded and the diagnosis of epilepsy will be set as per routine clinical practice at site. If standard approach is routine at site, patients will receive the

diagnosis of epilepsy after the onset of clinical or subclinical seizures. If preventive approach is routine at site, patients will receive diagnosis of epilepsy after the onset of epileptiform discharges on EEG meeting EPISTOP criteria (see Appendix 3).

Epileptogenesis will be tracked by means of serial video EEG (vEEG) recordings. vEEG will be performed every 4 weeks (± 1 week) in the children under the age of 6 months, then every 6 weeks (± 1 week) in children under 12 months, and every 8 weeks (± 1 week) thereafter, as recommended by the European guidelines for TSC infants (*Curatolo, 2012*). In patients whose parents/caregivers give consent for the randomized part of the project, EEG will be analysed by a local electroncephalographer who must be blinded to the clinical data of the patient. The EEG report must not be sent to the treating neurologist, but only to central randomizer. He will provide the treating neurologist with the diagnosis of epilepsy or not-epilepsy meaning that the patient should be treated or not, respectively. It means that if the treating neurologist receives a diagnosis of epilepsy, it is not known whether the patient had subclinical or clinical seizures recorded on videoEEG, or had electroencephalographic epileptiform discharges and was randomized to group diagnosed with epilepsy at that point. Similarly, if the treating neurologist receives a diagnosis of no epilepsy in a patient, it is not known whether EEG was normal, or the patient had epileptiform EEG, but was randomized to group diagnosed as epileptic at the onset of clinical seizures (Fig.3).

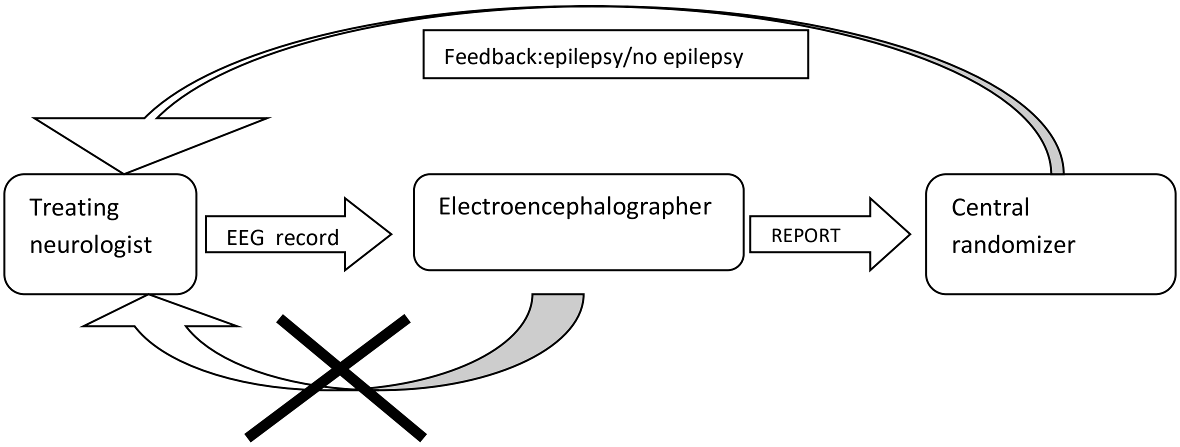

Fig.1.5. Scheme of study blinding: the treating neurologist will not know the results of EEG recordings. They will be reported by electroencephalographer blinded to the clinical data of the patient to the central randomizer. The treating neurologist will receive only the diagnosis: epilepsy or no epilepsy (meaning: treat or not treat, respectively).

In children with seizures, either noticed by a treating neurologist or caregivers or disclosed on vEEG, standard therapy with recommended first line antiepileptic drug will be given.

Children without seizures and no epileptiform discharges on vEEG will be followed without treatment. Infants that have epileptiform discharges on vEEG and no seizures will enter the randomized part of the study. Neuroimaging using magnetic resonance imaging (MRI) will be performed as recommended for TSC infants by European guidelines (*Curatolo, 2012*); specifically at baseline (unless patient had an MRI performed within 1 month before baseline visit and an additional scan cannot be performed without general anaesthesia) and then as clinically indicated, but not later than at the age of 2 years. In addition to standard MRI sequences, diffusion tensor imaging (DTI), diffusion-weighted imaging (DWI) will be performed as well as ASL sequences in children who were scanned on a 3T MRI without anesthesia. Neuropsychological assessment using a specially designed targeted battery of test will be performed every 6 months.

Blood samples for biomarker studies will be collected at study entry, at the onset of epileptiform discharges on vEEG or at the age of 6 months, whichever is applicable, at the onset of clinical seizures and at the end of follow-up (age 2 years) in all patients participating in the project. Blood sampling and storage of the samples details are provided in Appendix 7.

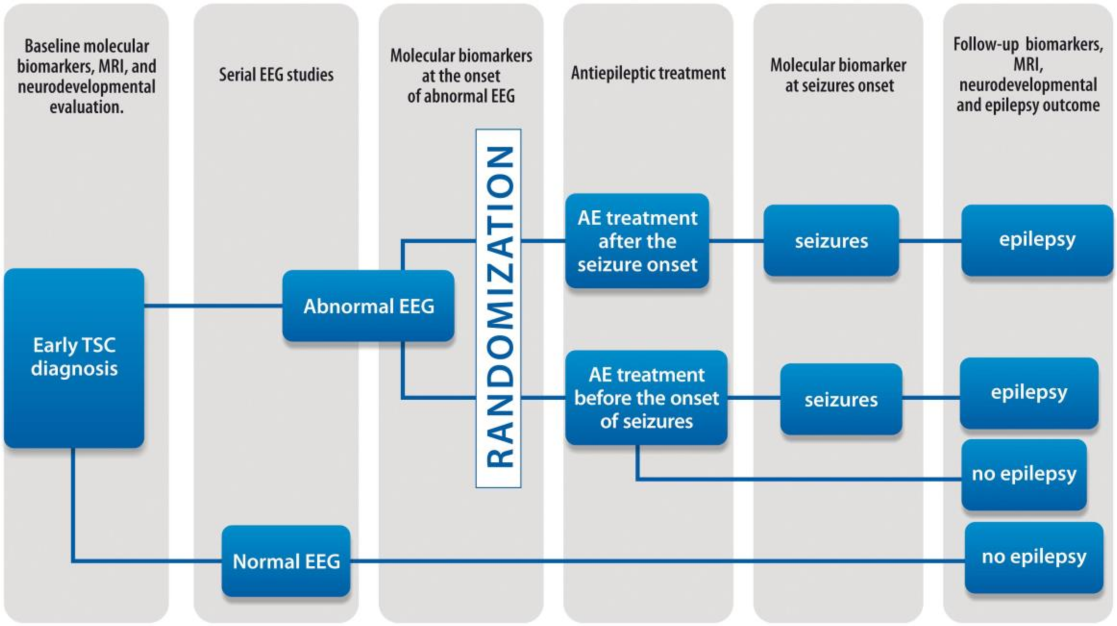

Fig.4. Flowchart of the clinical study.

Study procedures

Video EEG

Video EEG will be performed every 4 weeks in the children under the age of 6 months, then every 6 weeks in children under 12 months, and every 8 weeks thereafter. Video EEG will be recorded for at least 1 hour, including wake and sleep (up to stage 2). It is not allowed to use sedation to obtain sleep.

Video EEG will be performed using at least 19 electrodes according to the 10-20 system and one or two supplementary electrodes for ground and reference depending on the EEG recording system. For children under 3 months of age corrected age a reduced array can be considered.

Technical conditions of EEG recording include:

- electrode impedance should not exceed 5kOhm
- filter settings should be in the range of 1-70Hz
- high-pass filter should be no higher than 1Hz
- low-pass filter should be no lower than 70Hz

Sampling rate should be at least 250Hz. Simultaneous registration of 1 lead ECG (Lead II) and EMG (on both upper arms) should be performed.

Intermittent photic stimulation (IPS) as an activation procedure should be performed with at least 4 frequencies, with eyes open/closed (EO/EC) for background rhythm reactivity assessment. In non-cooperative and/or too young children, only EO recording will be performed.

Video EEG will be assessed both locally and centrally. Local video EEG reading should be performed immediately after the recording and should include the following assessments of the background, interictal epileptiform discharges, and seizures:

1. **Assessment of background:**

   BGN - background normal both in sleep and wake

   BGA - background abnormal in sleep and during wake

   BGAw - background abnormal only in wake

   BGAs - background abnormal only during sleep

2. **Distribution of abnormalities:**

   0 - no epileptic abnormality during wake and sleep

   A - 1 brain area with epileptic activity in one hemisphere (exception O1 and O2 together considered as 1 brain area)

B - >1 brain areas in one hemisphere with epileptic activity (non adjacent electrodes show interictal epileptic activity, or adjacent electrodes with IED but with clear temporal off-set)

C - Multifocal : 2 or more areas not in the same hemisphere

D - Generalized (or typical hypsarrhythmia)

3. **Severity of abnormalities:**

0 - no epileptic abnormality

I - very rare spikes , < 1% of time

II - epileptic activity for 1-10 % of the time

III - epileptic activity 10 - 50 % of the time

IV - epileptic activity > 50% of the time

V – Hypsarrhythmia

4. **Seizures**

NS - No seizures (NS)

S - Clinical seizure with concordant EEG changes

CS - clinical seizures only (video), no EEG correlate

SS - subclinical (electrographic) seizures, no video correlate

Criteria for randomization

Local electroencephalographist will assess vEEG according to the EPISTOP score and the score will be sent to the central randomizer (Prof. Lieven Lagae, KULeuven, Belgium) via e-mail or fax within 2 hours after video EEG recording. The central reader will identify patients who fulfil the criteria for randomization:

- no seizures (NS)

and one of the following findings on video EEG:

- distribution of abnormalities: A, and severity of abnormalities III
- distribution of abnormalities B, C, or D, irrespective of severity of abnormalities

MRI

MRIs will be performed on 1.5 or 3T MRI scanners, with minimum of 8-channel head coil. MRI sequences include:

- T1 SE 3mm
- T2 TSE_3D Vista
- DW/SSh
- T2 FLAIR 4mm

- T1W_3D_ISO
- 2.3x2.3x2.3 55sl 60gr 1NSA b1000
- ASL (only on 3Tesla)

MRIs will be performed under general anesthesia or with chloral hydrate and melatonine (6 mg < 1 year; 10mg > 1 year, 1 hour before scanning). The preferred method of sedation is chosen by the treating physician and should be according to the local routine clinical practice. It is important to use the same kind of sedation at baseline and 24 months (either both times general anesthesia or not), because general anesthesia influences ASL sequences. Therefore, we will only perform ASL sequences in a subgroup of patients that will not undergo general anesthesia (and are scanned on 3T scanners). All cooperating centres will be provided with an optimal centre specific protocol and optimize parameters for each scanner. All MRIs will be recorded on CD/DVD and send to the leader of work package 4 (dr. F.E. Jansen, UMCU, the Netherlands) for further analyses. Recorded images will only be labeled with the patient's code.

Every child enrolled in the study will undergo MRI at inclusion (before the age of four months). During follow up MRIs will be performed when clinically indicated or at least at the age of two years (end of study). This will give us the unique opportunity to follow longitudinally the MRI evolution in TSC, whether or not influenced by recurrent seizures.

Neuropsychological assessment

Neuropsychological assessment will be performed in each EPISTOP participant every 6 months, starting from baseline. The following tests will be performed:

1. The Bayley Scales of Infant Development (BSID): will be used to measure the child's level of development in three domains: cognitive, motor and behavioral. The test contains items specifically designed to identify young children at risk for developmental delay. The test is given on an individual basis and takes about 45-60 minutes to be completed. The time of assessment is at enrolment and every six months for a maximum of 4 times for 24 months (approximately at 6,12, 18 and 24 months according to the timing of enrolment).
2. The Autistic Diagnostic Observation Schedule (ADOS): will be used as the gold standard for assessing and diagnosing autism. The ADOS includes four modules, each requiring about 40 minutes to administer. The time of assessment is at enrolment and every six months for a maximum of 4 times for 24 months (approximately at 6,12, 18 and 24 months according to the timing of enrolment).

3. The Communication and Symbolic Behavior Scale Checklist (CSBSC): is a parent report checklist that measures 7 language/ communicative skill predictors. The checklist is to be completed by a caregiver and takes about 5-10 minutes to complete. It identifies children who have or are at-risk for developing communication impairment and monitors changes in a child's communication, expressive speech and symbolic behavior over time. The time of assessment is at 6, 12, 18 and 24 months.

The anonymous results of the tests will be labeled only with the patient's code and sent to WP7 leader (Prof. Paolo Curatolo, TVG, Italy) by e-mail (curatolo@uniroma2.it) for further analyses.

Blood biomarkers collection

Blood samples for biomarker studies will be collected at study entry, at the onset of epileptiform discharges on vEEG or at the age of 6 months, whichever is applicable, at the onset of clinical seizures and at the end of follow-up (age 2 years) in all patients participating in the project. Blood sampling and storage of the samples details are provided in Appendix 7.

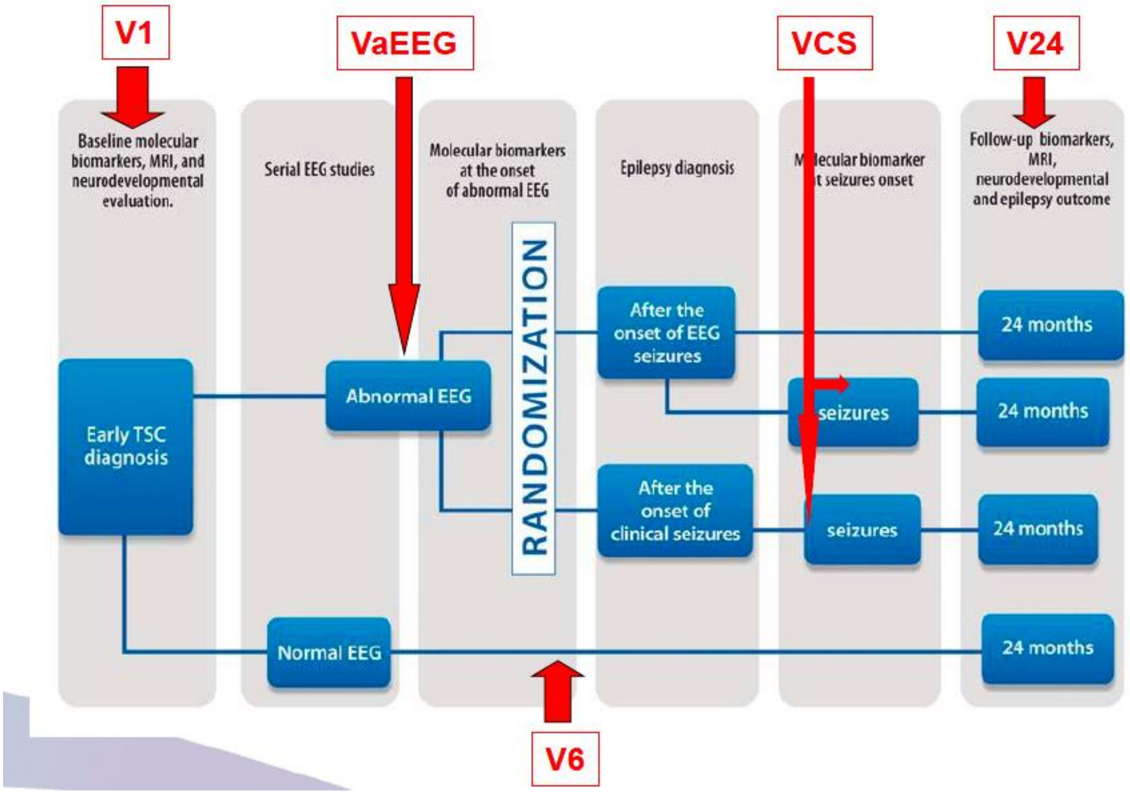

Fig.5. Blood sampling schedule.

Visit schedule

Baseline Visit

Prior to any protocol procedure, the patient's parents/caregivers must provide their informed consent by signing the Informed Consent Form. It must be clearly determined whether they

give consent for the observational part of the study only, or for the randomized part of the study. During screening visit, the medical history of the patient and patient's family will be collected. Full paediatric examination, including the recording of vital signs, and the assessment of neurodevelopmental status of the patient will be performed. First EEG recording will be performed and the score according to EPISTOP scale will sent to central reader. The patient should also have MRI performed (unless patient had an MRI performed within 1 month before baseline visit and an additional scan cannot be performed without general anaesthesia). First set of biomarkers from the blood should also be collected at baseline.

All data should be introduced into Case Report Form (CRF).

Next visits

Next visits should be scheduled every 4 weeks in the children under the age of 6 months, then every 6 weeks in children under 12 months, and every 8 weeks thereafter. On each visit, medical history of the patient should be reviewed and vEEG recording should be performed and the report sent to central reader. Seizure diary should be dispensed to patient's caregivers at each visit. If the patient is not diagnosed with epilepsy, next visit should be scheduled according to the age of the patient and no treatment should be given. If the patient is diagnosed with epilepsy, recommended first line antiepileptic drug should be started in standard dose. If the patient is already on recommended first line antiepileptic drug and still experiences seizures, then second antiepileptic drug should be introduced according to the expertise and opinion of the treating physician. At the age of 6 months, if not taken earlier, the second set of biomarkers should be taken from the blood. Third set of biomarkers from the blood should be taken at the onset of clinical seizures. If the patient starts experiencing seizures, a seizure diary should be dispensed, collected and reviewed at every visit. Neuropsychological examination should be performed every 6 months and at the follow-up visit, at the age of 24 months in each patient. All data should be entered into CRF.

Follow-up visit

Follow-up visit should be scheduled at the age of 24 months. At that visit, the detailed medical history, including epilepsy details and medications, should be reviewed. Full paediatric examination, neurodevelopmental assessment, neuropsychological examination, last vEEG, and MRI should also be performed. At follow-up visit, the last set of blood biomarkers should be taken. In TSC infants that develop epileptiform discharges on vEEG, and are randomized to have the diagnosis of epilepsy resulting with treatment, and never

develop clinical seizures, antiepileptic drug will be tapered off starting from the follow-up visit.

Patients' numbering

Each patient will be identified in the study by code, that will be assigned during baseline visit. This code will be the primary identifier of the patient throughout the study. The code will consist of site identifier and sequential patient number (for example: 01-001). Once assigned, the patient's code cannot be re-used or changed.

Site identifying numbers:

**Site**	**Number**
The Children's Memorial Health Institute	01
Tor Vergata University	02
Inserm; Hopital Necker Enfants Malades	03
Universitair Ziekenhuis Brussel (UZ Brussels)	04
University Hospital Motol, Charles University	05
Charité – University Medicine Berlin	06
University Medical Center, Utrecht	07
University Hospital Leuven	08
Medical University of Vienna	09
Lady Cilento Children's Hospital, Brisbane	16

Randomization procedures

Patients with epileptiform discharges on vEEG noted prior to seizures will enter the randomized part of the study. The time point of randomization will not be known to the treating neurologist to keep the study blinded. Central randomizer will provide the diagnosis of the patient to the treating neurologist after each EEG recording.

Randomization will be performed as block randomization stratified for centre. Patients will be randomized in ratio 1:1.

Adverse events

An Adverse Event (AE) is any adverse change from the patient's baseline condition that occurs during the course of the study, irrespective to the relation to the epilepsy approach.

However, **it is important that this study does not test any investigational product**, so only the undesirable effects of epilepsy diagnosis before or after the onset of seizures should be considered as study-related (and not drug-related, as there is no investigational drug).

Adverse events do not include:

- epileptic seizures, unless worsened due to late epilepsy diagnosis;
- planned medical or surgical procedures, ie. vaccinations, hospitalizations related to control examinations, like cardiologic follow-ups etc.
- pre-existing disease or medical condition that does not worsen,

However, these events should be mentioned in eCRF (in Others or Comments section in the respective visit record). Seizures must be reported on dedicated eCRF sections. Parents of patients experiencing seizures should be asked to report the number and type of seizures daily and these data should be transferred to eCRF.

For each AE, the relation to the study should be assessed by the investigator and reported in eCRF. If the causal relationship between the AE and the project is possible or certain, the relevant comment including the justification must be recorded on eCRF.

Serious Adverse Events (SAE) is any AE fulfilling any of the following criteria:

- fatal
- life-threatening
- requiring hospitalization or prolongation of existing hospitalization, with the exception of planned hospitalizations
- resulting in persistent or significant disability
- medically significant or requiring intervention to prevent any of the outcomes listed above

All SAE regardless their relation to the study must be recorded in eCRF and reported to the Project Coordinator (Sergiusz Jóźwiak, at IPCZD) within 48 hours by fax: **+48 22 815 74 02** or e-mail: **sergiusz.jozwiak@gmail.com** or WP6 learder Prof. Katarzyna Kotulska (e-mail: k.kotulska@czd.pl). The coordinator will contact appropriate health authorities as well as Scientific Advisory Board and Ethics Committee. All SAEs must be followed until resolution or stabilization. Follow up report must be recorded within 30 days after the onset of SAE or earlier, if possible, and reported to the Study Coordinator by fax or e-mail.

Data collection and management

Clinical data will be captured using electronic Case Report Form (eCRF). Data will be documented in various source documents and then manually entered into the eCRF by study

site personnel. eCRF will be provided to each site by IPCZD.

All information about study subjects will be confidential and managed according to local regulations and laws. Specifically, a signed authorization of the patient's caregivers informing of the following is required:

- what protected health information will be collected from the patients in this study
- who will have access to that information and why
- who will use or disclose that information
- the rights of the study subject caregivers to revoke their authorization for use of their protected health information.

The patient's caregivers will be told that representatives of the Consortium, ethics committees, and regulatory authorities may inspect their medical records to verify the information collected. They will also be told that all personal data available for the inspection will be handled in strictest confidence and in accordance with local data protection laws.

In case that a patient's caregivers revoke their authorization to use or to collect protected health information, only information collected prior to the revocation of the authorization can be used.

Following the study completion, all documents related to the project, including patients' source data, should be stored on site according to local legal regulations.

Monitoring plan

Monitoring will be performed by monitor (CRA) chosen by IPCZD, coordinating site. In each participating centre one monitoring visit will be conducted annually.

Verification of source data

- 100% check of presence and correctness of Informed Consent Forms
- 100% check of inclusion and exclusion criteria
- 50% check of source documents of the following study data:
    - Age
    - Sex
    - Diagnosis TSC
    - Diagnosis allocation
    - Date of inclusion
    - Result of MRI, EEG and neuropsychological tests
    - Seizures diary
- 100% check of SAE's as well as verification of the appropriate reporting procedures

General control

- For each visit of each centre the rate of inclusion and the dropout rate will be reported.
- For each centre study procedures will be checked as well as the ability of the study personnel to comply with these procedures.

Reporting

The monitor will provide a written report to the Coordinator after each visit to a study location. This report will be stored by the Coordinator and will be directly available for an audit. A study location will receive a written summary of the control procedures that have been performed and the associated findings.

The 'monitor visit report' will include:

- A summary of control procedures performed by the monitor
- A general description of quality at the study site
- A summary stating the most important findings / facts, deviations and shortcomings
- An overview of proposed measures and recommendations to ensure compliance with the protocol
- The general conclusion

The Coordinator will receive the originals of initiation visit report and the close-out visit report and principal investigator of each site will receive copies of these documents. If applicable, other relevant contacts considering the study will be enclosed as a written report.

Data quality assurance

The eCRFs and other essential documents will be reviewed by a clinical monitor designed by IPCZD.

Essential documents include:

- signed informed consent documents for all subjects
- the decision of ethics committee together with the composition of ethics committee
- records of all communications between the investigator and the ethics committee
- all source documents (patient records, hospital records, laboratory records, seizure diaries, etc)
- any other documents required by local laws or GCP guidelines.

Data on CRFs will be source-verified during site visits for accuracy and completeness according to the monitoring plan. The visits will take place annually as the minimum. The first visit will be scheduled 12 months after the first visit of the first patient enrolled by the site. The investigator will allocate adequate time for such monitoring activities. The

investigator will also ensure that the monitor is given access to all above noted study documents and has adequate space to conduct the monitoring visit.

The safety of patients in the study will be monitored by Independent Ethics Board supported by a biostatistician designated by IPCZD. The Independent Ethics Board will perform the review of the safety and efficacy after 70% of all planned randomized patients complete the study and their seizures outcome data are available.

Early stopping rules

The randomized part of the study can be stopped early if the external Scientific Advisory Board together with Steering Committee decides so, based on the analysis of study safety and/or efficacy. Particularly, the study can be stopped for unacceptable safety. In this case, all participating and new patients will enter the observational part of the project only.

Second, the study may be stopped if any of the compared epilepsy approaches (early diagnosis versus diagnosis after clinical seizures) shows clear benefit in terms of seizure outcome, evidenced by the interim statistical analysis. Seizure outcome includes the number of seizure-free patients, the number of patients with normalized EEG, and the number of patients with drug-resistant epilepsy. The interim analysis will be performed when 70% of all planned randomized patients complete the study and their seizures outcome data are available. The statistical significance of P-value of 0.01 at interim analysis as evidence to stop early for benefit is required. In this case, all new patients and patients already participating in the study but not having abnormal EEG yet, will be offered the approach that was proved to be significantly more efficient. Other patients already participating in the study will continue their treatment.

Withdrawal of individual subjects

Subjects can leave the study at any time for any reason if they wish to do so without any consequences. The investigator/ treating physician can decide to withdraw a subject from the study whenever he/she considers that continuation in the study would adversely affect the subjects' health.

Statistical analyses

Full analysis set comprises all patients participating in the study, including the control group. This set will be divided into subsets: control group, TSC patients with epilepsy, and TSC patients with no epilepsy. Among TSC patients with epilepsy, patients with well-controlled seizures and patients with drug-resistant epilepsy will be identified. In full analysis set the

blood biomarkers will be analysed. Clinical analysis set will comprise of all TSC infants enrolled in the study and the clinical biomarkers of epileptogenesis (neuroimaging, vEEG, data from medical history) will be analysed in this set. Treatment analysis set will comprise of infants participating in the randomized part of the study and the efficacy of antiepileptic treatment in respect to the point of epilepsy diagnosis (electroencephalographic epileptiform discharges onset in group A and seizures onset in group B) will be assessed in this set.

The interim analyses will be performed when 70% of the patients will complete the whole study. Final analyses will be performed when the last patient will complete the study (at the age of 24 months).

Regarding clinical outcome measures and the study endpoints, the sample size was determined based on our previous study (*Jozwiak, 2011*), in which similar parameters were assessed. **The statistical analysis** will include two types of tests:

- qualitative variables (frequencies of occurrence) will be analysed using chi-square tests
- quantitative variables using non-parametric equivalencies of ANOVA tests.

The power of these tests was calculated for the p-level (alpha error) set at 0.5, and assuming the balanced distribution of patients in the groups. As described, we expect that 60-70% of subjects will have epileptiform EEG changes prior to onset of seizures, and thus be randomized to either A or B group. We predict that early diagnosis and treatment will be 50-60% effective in preventing clinical seizure development in the patients that are randomized to group A. Given a set of 60 patients, with 30 going to group A and 30 to group B, our power to detect this difference in clinical seizure development is higher than 80%. All calculations were done using G*Power v 3.1.3 freeware, Kiel University, Germany.

Ethical considerations

EPISTOP project was designed and shall be implemented and reported in accordance with ICH Harmonized Tripartite Guidelines for Good Clinical Practice and the following EU legislations:

- The Charter of Fundamental Rights 2000 of the EU;
- European Directives 95/46/EC, 2002/58/EC, and 2001/20/EC.

All participants in EPISTOP will respect the ethical principles laid down by national regulations and the following international conventions and declarations:

- Helsinki Declaration;
- Oviedo Convention of the CE on Human Rights and Biomedicine;

- UN Convention on the Rights of Child;
- Universal Declaration on the human genome and human rights adopted by UNESCO.

This clinical study was designed, shall be implemented and reported in accordance with Good Clinical Practice, the regulations given in EU Directive No 20/2001 and other relevant regulations to ensure patients' safety. We will use a unified electronic Case Report Form to ensure the quality of data entered into the database, and an external data monitor (Clinical Research Associate; CRA) to monitor the progress of the study. Ethics issues will be supervised also by the external Scientific Advisory Board and Ethical Committee.

EPISTOP has been already accepted by the local Ethics Board at the Children's Memorial Health Institute (IPCZD). This opinion of the Ethics Board is provided as an Appendix 9.

Informed consent procedures

Eligible children may only be included in the study after their legal representatives (parents, caregivers) provide their Ethical Committee – approved informed consent. The English, basic version of the informed consent forms will be translated into national languages. The national versions will be subjected to local Ethical Committees opinion and used only after having approval.

Informed consent must be obtained before conducting any study-specific procedures. The participants/their representatives should have the opportunity to discuss all the procedures, possible risks and benefits with the study team. They will be given enough time to read and understand the information form and ask any clarifications.

One copy of the signed informed consent form should be stored at site files and the second one should be given to the participant/legal representatives of the participant. The process of obtaining informed consent should be reported in the patient's chart.

Personal data protection

EPISTOP will follow the principles for the protection of personal data laid down by European legal regulations. The study coordinator will ensure that consent for data storage is obtained from all participants and the data will be used only if there is consent for its use. Study subjects genetic, epigenetic, biochemical, proteomic, electroencephalographic, neuroimaging, and other medical data will be stored and analyzed in a coded/anonymous form. Data management and data safety reports will be regularly presented to the Advisory Board.

Electronic case report form will be used for web-based data entry. The data entered into this database will be de-identified and only the responsible researchers will have the access to identifying details.

The collaborative nature of EPISTOP requires the exchange of information and biological material between European and US partners. For purposes of WP3, WP5, WP7, biological samples obtained from patients and the relevant medical data will be transferred between sites. Only de-identified and coded samples as well as necessary medical data will be sent. The special safety provisions concerning biological material transportation will be considered and has been taken into account when calculating the costs of the project.

The participants of EPISTOP and/or their legal representatives will be informed that they have the right to cancel their consent at any time by giving written notice to investigator. If the consent is cancelled, then the investigators will no longer use or disclose any medical information of the participant. However, canceling this consent will not affect previous uses and disclosures and the already existing participant's medical information would not be removed from the study records.

The data produced in this study will be stored in a locked, secure location. Only members of the research team will have access to this location. Following completion of the research study the data will be kept as long as required by law and then destroyed as required by the hospital, laboratory or institute policy.

EPISTOP assures that the key design elements of the project will be posted in its publicly accessible website. The results of the project will be submitted to publication in medical journals with the respect of participants' personal data protection.

References

Blumenfeld H, Klein JP, Schridde U, et al. Early treatment suppresses the development of spike-wave epilepsy in a rat model. Epilepsia 2008; 49(3): 400-409.

Bombardieri R, Pinci M, Moavero R, Cerminara C, Curatolo P. Early control of seizures improves long-term outcome In children with tuberous sclerosis complex. Eur J Paediatr Neurol 2010; 14(2): 146-149.

Camposano SE, Major P, Halpern E, Thiele EA. Vigabatrin in the treatment of childhood epilepsy: a retrospective chart review of efficacy and safety profile. Epilepsia; 2008; 49(7): 1186-91.

Chiron C, Dulac O. Epilepsy: Vigabatrin treatment and visual field loss. Nat Rev Neurol. 2011; 7(4): 189-90.

Curatolo P, Jóźwiak S, Nabbout R. TSC Consensus Meeting for SEGA and Epilepsy Management. Management of epilepsy associated with tuberous sclerosis complex (TSC): clinical recommendations. Eur J Paediatr Neurol. 2012; 16(6):582-6.

Durbin S, Mirabella G, Buncic JR, i wsp. Reduced grating acuity associated with retinal toxicity in children with infantile spasms on vigabatrin therapy. Invest Ophthalmol Vis Sci. 2009 Aug;50(8):4011-6.

Fastenau PS, Johnson CS, Perkins SM, et al. Neuropsychological status at seizure onset in children: risk factors for early cognitive deficits. Neurology 2009; 73(7): 526-534.

Greiner HM, Lynch ER, Fordyce S, et al. Vigabatrin for childhood partial-onset epilepsies. Pediatr Neurol, 2012; 46(2): 83-88.

Hammoudi DS, Lee SS, i wsp. Reduced visual function associated with infantile spasms in children on vigabatrin therapy. Invest Ophthalmol Vis Sci. 2005 Feb;46(2):514-20

Howie SR. Blood sample volumes in child health research: review of safe limits. Bull World Health Organ. 2011; 89(1): 46-53.

Jóźwiak S, Kotulska K, Domańska-Pakieła D, et al. Antiepileptic treatment before the onset of seizures reduces epilepsy severity and risk of mental retardation in infants with tuberous sclerosis complex. Eur J Paediatr Neurol. 2011;15(5):424-31.

Philippi H, Wohlrab G, Bettendorf U, et al. Electroencephalographic evolution of hypsarrhytmia: toward an early treatment option. Epilepsia 2008; 49(11): 1859-1864.

Primec ZR, Stare J, Neubauer D. The risk of lower mental outcome in infantile spasms increases after three weeks of hypsarrhythmia duration. Epilepsia 2006; 47: 2202-2205.

van Rooij L, Toet M, van Huffelen A, et al. Effect of treatment of sublinical neonatal seizures detected with aEEG: randomized, clinical trial. Pediatrics, 2010; 125: e358.

Appendices

Appendix 1: Names and contact details of cooperating sites

Appendix 2: Overview of work packages

Appendix 3: Video EEG protocol

Appendix 4: MRI protocol

Appendix 5: Neuropsychological assessment protocol

Appendix 6: Patient's seizure diary

Appendix 7: Blood sampling protocol

Appendix 8: Sample handling form

Appendix 9: EC approval Warsaw, Poland

Appendix 10: EC approval Rome, Italy

Appendix 11: EC approval Prague, Czech Republic

Appendix 1

Coordinating Center

1. Instytut "Pomnik-Centrum Zdrowia Dziecka" (IPCZD) / The Children's Memorial Health Institute
   Al. Dzieci Polskich 20
   04-730 Warszawa
   Poland
   Prof. Sergiusz Jozwiak
   T: +48 22 815 74 04
   E: sergiusz.jozwiak@czd.pl

Partners:

2. Universita Degli Studi Di Roma Tor Vergata (TVG)
   Via Orazia Raimondo
   18 00173 Rome
   Italy
   Prof. Paolo Curatolo
   T: +39 0 62 0 90 02 49
   E: curatolo@uniroma2.it

3. Institut National De La Sante et de la Recherche Medicale (INSERM)
   149 Rue de Sevres
   75015 Paris
   France
   Prof. Rima Nabbout
   T: +33 14 438 15 36
   E: rimanabbout@yahoo.com

4. Vrije Universiteit Brussel (VUB)
   Pleinlaan 2
   1050 Brussel
   Belgium
   Prof. Anna Jansen
   T: +32 2 477 57 85
   E: anna.jansen@vub.ac.be

5. Fakultni Nemocnice V Motole (UHM)
   V Uvalu 84
   15006 Praha 5
   Czech Republic
   Dr. Pavel Krsek
   T: +42 022 443 33 51 (3300)
   E: pavel.krsek@post.cz

6. Charite – Universitaetsmedizin Berlin (CUB)
   Chariteplatz 1
   D-10117 Berlin

Germany
Dr. Bernhard Weschke
T: +49 30 450 666 617
E: bernhard.weschke@charite.de

7. Universitair Medisch Centrum Utrecht (UMC)
Lundlaan 6
3584 EA Utrecht
Postbus 85090
3508 GA Utrecht
Netherlands
Dr. Floor Jansen
T: +31-88 755 4341
E: f.e.jansen@umcutrecht.nl

8. Katholieke Universiteit Leuven (KU Leuven)
Waaistraat 6
Box 5105
3000 Leuven
Belgium
Prof. Lieve Lagae
T: 32 16 34 38 45
E: lieven.lagae@uzleuven.be

9. Medizinische Universitaet Wien (MUW)
Spitalgasse 23
1020 Wien
Austria
Prof. Martha Feucht
T: 43 14 0 400 38 05
E: martha.feucht@meduniwien.ac.at

10. Academisch Medisch Centrum Amsterdam (AMC)
Meibergdreef 9
1105 AZ Amsterdam
Netherlands
Prof. Eleonora Aronica
T: 31 20 566 95 22
E: e.aronica@amc.uva.nl

11. International Institute of Molecular and Cell Biology (IIMCB)
ul. Ks. Trojdena 4
02-109 Warszawa
Poland
Prof. Jacek Jaworski
T: +48 22 5970 755
E: jaworski@iimcb.gov.pl

12. The Brigham and Women's Hospital (BWH)
Francis Street 75

02 115 Boston
USA
Dr. David Kwiatkowski
T: +1 617 355 9005
E: djk@partners.org

13. Proteome Factory AG (PFA)
Magnusstrasse 11
D-12489 Berlin
Germany
Prof. Christian Scheler
T: +49 302 061 6265
E: scheler@proteomefactory.com

14. Service XS (SXS)
Plesmanlaan 1d
2333 BZ Leiden
Netherlands
Prof. Bart Janssen
T: +31 715 68 1018
E: b.janssen@servicexs.com

15. A.R.S consulting s.r.l (ARS)
Via S. Grandis, 1-00185 Rome
Italy
Alberto Sciuto
T: +39067014680
E: alberto.sciuto@arsconsulting.it

16. Lady Cilento Children's Hospital
Neurosciences Unit, level 6C
501 Stanley Street
Brisbane QLD4101, Australia
Dr. Kate Riney
T: +61730682880
E: Kate.Riney@health.qld.gov.au

Appendix 2

WP1	Project and database management	IPCZD
WP2	EEG tracking of epilepsy and epileptogenesis in TSC patients	KU Leuven
WP3	Identification of molecular biomarkers of epilepsy risk and epileptogenesis in TSC patients	SXS, AMC, BWH
WP4	Neuroimaging findings as biomarkers of epilepsy risk and epileptogenesis in TSC patients	UMC
WP5	Validation of biomarkers of epilepsy and molecular targets for novel therapies in human TSC specimen	AMC
WP6	Clinical study of epileptogenesis and randomized preclinical epilepsy diagnosis in TSC patients	IPCZD
WP7	Identification of the prognostic factors of neurodevelopmental outcome in TSC patients	TVG
WP8	Exploitation and dissemination	VUB

Appendix 3

Video EEG

Video EEG will be performed every 4 weeks in the children under the age of 6 months, then every 6 weeks in children under 12 months, and every 8 weeks thereafter. Video EEG will be recorded for at least 1 hour, including wake and sleep (up to stage 2). It is not allowed to use sedation to obtain sleep.

Video EEG will be performed using at least 19 electrodes according to the 10-20 system and one or two supplementary electrodes for ground and reference depending on the EEG recording system. For children under 3 months of age corrected age a reduced array can be considered.

Technical conditions of EEG recording include:

- electrode impedance should not exceed 5kOhm
- filter settings should be in the range of 1-70Hz
- high-pass filter should be no higher than 1Hz
- low-pass filter should be no lower than 70Hz

Sampling rate should be at least 250Hz. Simultaneous registration of 1 lead ECG (Lead II) and EMG (on both upper arms) should be performed.

Intermittent photic stimulation (IPS) as an activation procedure should be performed with at least 4 frequencies, with eyes open/closed (EO/EC) for background rhythm reactivity assessment. In non-cooperative and/or too young children, only EO recording will be performed.

Video EEG will be assessed both locally and centrally. Local video EEG reading should be performed immediately after the recording and should include the following assessments of the background, interictal epileptiform discharges, and seizures (EPISTOP epilepsy scale):

1. **Assessment of background:**

- **BGN** - background normal both in sleep and wake
- **BGA** - background abnormal in sleep and during wake
- **BGAw** - background abnormal only in wake
- **BGAs** - background abnormal only during sleep

2. **Distribution of abnormalities:**

   0 - no epileptic abnormality during wake and sleep

   A - 1 brain area with epileptic activity in one hemisphere (exception O1 and O2 together considered as 1 brain area)

B - >1 brain areas in one hemisphere with epileptic activity (non adjacent electrodes show interictal epileptic activity, or adjacent electrodes with IED but with clear temporal off-set)

C - Multifocal: 2 or more areas not in the same hemisphere

D - Generalized (or typical hypsarrhythmia)

3. **Severity of abnormalities:**

   0 - no epileptic abnormality

   I - very rare spikes, < 1% of time

   II - epileptic activity for 1-10 % of the time

   III - epileptic activity 10 - 50 % of the time

   IV - epileptic activity > 50% of the time

   V – Hypsarrhythmia

4. **Seizures**

   NS - No seizures (NS)

   S - Clinical seizure with concordant EEG changes

   CS - clinical seizures only (video), no EEG correlate

   SS - subclinical (electrographic) seizures, no video correlate

Criteria for randomization

Local electroencephalographist will assess vEER records according to EPISTOP scoring system and the result of the analysis will be sent to the central reader (Prof. Lieven Lagae, KULeuven, Belgium) via e-mail or fax within 2 hours after video EEG recording. The software will enable identification of patients who fulfil the criteria for randomization:

- no seizures (NS)

and one of the following findings on video EEG:

- distribution of abnormalities: A, and severity of abnormalities III
- distribution of abnormalities B, C, or D, irrespective of severity of abnormalities

All sent data will be anonymous and labelled with patients code only.

Appendix 4

MRI

MRIs will be performed on 1.5 or 3T MRI scanners, with minimum of 8-channel head coil. MRI sequences include:

- T1 SE 3mm
- T2 TSE_3D Vista
- DW/SSh
- T2 FLAIR 4mm
- T1W_3D_ISO
- 2.3x2.3x2.3 55sl 60gr 1NSA b1000
- ASL (Orly on 3Tesla)

MRIs will be performed under general anesthesia or with chloral hydrate and melatonin (6 mg < 1 year; 10mg > 1 year, 1 hour before scanning). The preferred method of sedation is chosen by the treating physician and should be according to the local routine clinical practice. It is important to use the same kind of sedation at baseline and 24 months (either both times general anesthesia or not), because general anesthesia influences ASL sequences. Therefore, we will only perform ASL sequences in a subgroup of patients that will not undergo general anesthesia (and are scanned on 3T scanners).
We will provide all cooperating centers with an optimal center specific protocol and optimize parameters for each scanner. All MRIs will be recorded on CD/DVD and send to the leader of work package 4 (dr. F.E. Jansen, UMCU) for further analyses. Recorded images will only be labeled with the patient’s code. Every child enrolled in the study will undergo MRI at inclusion (before the age of four months). During follow up MRIs will be performed when clinically indicated or at least at the age of two years (end of study). This will give us the unique opportunity to follow longitudinally the MRI evolution in TSC, whether or not influenced by recurrent seizures.

Appendix 5

Neuropsychological assessment will be performed in each EPISTOP participant every 6 months, starting from baseline. The following tests will be performed:

1. The Bayley Scales of Infant Development (BSID): will be used to measure the child's level of development in three domains: cognitive, motor and behavioral. The test contains items specifically designed to identify young children at risk for developmental delay. The test is given on an individual basis and takes about 45-60 minutes to be completed. The time of assessment is at enrolment and every six months for a maximum of 4 times for 24 months (approximately at 6,12, 18 and 24 months according to the timing of enrolment).

2. The Autistic Diagnostic Observation Schedule (ADOS): will be used as the gold standard for assessing and diagnosing autism. The ADOS includes four modules, each requiring about 40 minutes to administer. The time of assessment is at enrolment and every six months for a maximum of 4 times for 24 months (approximately at 6,12, 18 and 24 months according to the timing of enrolment).

3. The Communication and Symbolic Behavior Scale Checklist (CSBSC): is a parent report checklist that measures 7 language/ communicative skill predictors. The checklist is to be completed by a caregiver and takes about 5-10 minutes to complete. It identifies children who have or are at-risk for developing communication impairment and monitors changes in a child's communication, expressive speech and symbolic behavior over time. The time of assessment is at 6, 12, 18 and 24 months.

The anonymous results of the tests will be labeled only with the patient's code and sent to WP7 leader by email (Prof. Paolo Curatolo, curatolo@uniroma2.it) for further analyses.

Appendix 6

Patient's Seizure Diary

Site………………………………………………………….

Patient initials……………………… Patient number…………………

date of visit………………………………

Date	Number of seizures	Type of seizures (describe)	Antiepileptic drugs changes (including rescue medications)

Appendix 7

Blood sampling protocol

Title of Research Project:

Long-term, prospective study evaluating clinical and molecular biomarkers of epileptogenesis in a genetic model of epilepsy – tuberous sclerosis complex.

Project acronym: EPISTOP

A. Samples to be taken at certain time points:

1. **At baseline**: two RNA samples (V1 RNAa and V1 RNAb), one serum sample, divided into 5 portions (V1 Sa-e), one DNA sample (if possible; if the child is too young, this sample can be taken at any time point)
2. **At the onset of epileptiform abnormalities on EEG**: two RNA samples (VaEEG RNAa and VaEEG RNAb), one serum sample, divided into 5 portions (VaEEG S2a-e), one DNA sample (if not taken earlier)

   or

   At the age of 6 months: two RNA samples (V6 RNAa and V6 RNAb), one serum sample, divided into 5 portions (V6 S2a-e), one DNA sample (if not taken earlier)

3. **At the onset of clinical seizures**: two RNA samples (VCS RNAa and VCS RNSb), one serum sample, divided into 5 portions (VCS S3a-e), one DNA sample (if not taken earlier)
4. **At age of 24 month**s: two RNA samples (V24 RNAa and V24 RNAb), one serum sample, divided into 5 portions (V24 S4a-e), one DNA sample (if not taken earlier)

PROCEDURE

UNIVERSAL PRECAUTIONS: *Any biological samples derived from patients are considered to be potentially biohazardous. Utilize appropriate precautions when working with human-derived samples (i.e. personal protection equipment such as gloves, lab coat, and safety glasses). All waste (samples and related contact materials) must be placed in marked biohazardous waste containers and disposed according to your hospital guidelines.*

NOTE: *Best results are obtained when whole blood is processed promptly.*

NOTE: *All tubes should be appropriately labelled with patient's identification and date of processing. Please use a label maker.*

NOTE: *Manipulation to be performed under aseptic conditions.*

Samples labeling:

Please label the tubes with the patient's number (number of the site followed by sequential number of the patient), patient initials, the date (DD-MON-YEAR) and the visit identification number as follows:

Visit	Visit Identification number
Baseline	V1
Abnormal EEG	VaEEG
Clinical seizures	VCS
Age of 6 months	V6
Age of 24 months	V24

For example, the code for baseline visit at 01 JAN 2014 of the patient A-A, number 01-001 will be:

01- 001 A-A
V1 01-JAN-2014

Fill the missing data on the prepared stickers and print them.

RNA sampling:

Sample handling form:

Please fill this form for each patient prior to blood sampling.

Time of blood collection:

The sample can be taken at any time. Any fasting prior to blood draw is not necessary.

For Blood Collection:

Two Qiagen Paxgene RNA 2.5mL tubes. The 10 mL tubes contain 7,5 ml buffer, leaving space for 2.5 mL blood.

Vacutainer device

Blood Processing:

Fill both tubes with 2.5mL of blood (each).

Blood RNA samples can be stored in the fridge for 2-3 days (temporary storage) and then transferred to -70 or -80 degrees Celsius freezer for long-term storage. Alternatively, transfer serum samples directly to -70 or -80 degrees Celsius.

DNA sampling:

Sample handling form:

Please fill this form for each patient prior to blood sampling.

Time of blood collection:

The sample can be taken at any time. Any fasting prior to blood draw is not necessary.

For Blood Collection:

Qiagen Paxgene Blood DNA tube.

Vacutainer device

Blood Processing:

Fill the tube with 3 mL of blood.

Blood samples can be stored at room temperature for maximum 14 days. Alternatively, they can be sent frozen immediately in -20 degrees Celsius.

Serum sampling:

Sample handling form:

Please fill this form for each patient prior to blood sampling.

Time of blood collection:

The sample can be taken at any time. Any fasting prior to blood draw is not necessary.

For Blood Collection:

4 Starstedt 2.7 mL tubes

Vacutainer device

5 prelabeled cryovials

Transfer pipettes

Blood Processing:

Fill the each tube with 2.5 – 2.5 mL of blood to have 9 mL of blood. Keep the tubes standing upright for 30 minutes to allow blood to clot. This should be done at room temperature and away from direct light source. Remove the clot by centrifuging for 10 min at 2500 rpm on centrifuge with swinging bucket rotor with brake on full**. Keep the clot frozen and sent it together with serum to the central lab!**

The resulting supernatant is designated serum. Following centrifugation, immediately transfer the liquid component (serum) into the cryovials dividing it into 1 mL aliquots into cryovial a and b, 1.5 mL into cryovial c, 0.5 mL into cryovial d, and the rest into cryovial e, using a Pasteur pipette. The samples should be maintained at 2-8°C while handling.

**Cryovial label**	**Volume to fill**
**a**	**1mL**
**b**	**1mL**
**c**	**1.5 mL**
**d**	**0.5 mL**
**e**	**Remaining volume**<br><br>**(please record)**

Serum samples can be frozen at -20 degrees Celsius for not more than 2-4 hours (temporary storage) and then transferred to -70 or -80 degrees Celsius freezer for long-term storage. Alternatively, transfer serum samples directly to -70 or -80 degrees Celsius.

SAMPLE STORAGE INSTRUCTIONS:

Store all cryovials at –70°C OR -80°C in storage boxes.

All cryovials from the same participant should be stored together (not separated).

SAMPLE SHIPPING INSTRUCTIONS:

All samples should be shipped to the central lab (see below). The samples should be sent in dry ice.

Please arrange shipment every 3 months.

<u>Important: Avoid shipping on Fridays!</u>

Details regarding shipping will be provided soon.

CENTRAL LAB:

All samples will be stored at the central laboratory at the Laboratory of Molecular and Cellular Neurobiology; International Institute of Molecular and Cell Biology, ul. Trojdena 4, 02-109 Warsaw, Poland. The responsible person will be prof. Jacek Jaworski, e-mail: jaworski@iimcb.gov.pl. The samples will be stored deep-frozen, for up to 10 years, and will be destroyed after this period by the central laboratory.

Correspondence address:

Jacek Jaworski/Magdalena Blazejczyk/Aleksandra Piechnik

International Institute of Molecular and Cell Biology

ul. Trojdena 4

02-109 Warszawa

POLAND

phone: +48 22 597 07 55 or +48 22 597 07 57

e-mail: **epistop_shipment@iimcb.gov.pl**

Important: When filling the shipping list please remember to include all three names of recepients mentioned above. It will enable to pick up the package in case one person is absent.

Appendix 8

Sample handling form

Site No…………………..

Patient No………………

Patient initials…………..

Date of visit…………….. (DD MON YEAR)

Visit:

□ V1 □ VaEEG □ VCS □ V6 □ V24

Sampes taken:

□ RNAa □ RNAb

□ Sa □ Sb □ Sc □ Sd □ Se (….mL)

□ DNA

Important comments:

Date…………………………

Investigator's name………………… Signature………………………..

Appendix 9

[m.p.]
KOMISJA BIOETYCZNA
przy Instytucie "Pomnik-Centrum Zdrowia Dziecka"
04-730 Warszawa, Al. Dzieci Polskich 20
fax/tel. +48 22 8157571; tel. +48 22 8157572
e-mail: komisja.bioetyczna@czd.pl

UCHWAŁA nr 66/KBE/2013

OPINIA KOMISJI BIOETYCZNEJ
przy INTYTUCIE „POMNIK-CENTRUM ZDROWIA DZIECKA"

Komisja Bioetyczna przy Instytucie „Pomnik-Centrum Zdrowia Dziecka" **na posiedzeniu w dniu 23.01.2013 r.** rozpatrzyła projekt badania pt.: **Długoterminowe, prospektywne badanie oceniające kliniczne i molekularne biomarkery epileptogenezy w modelu padaczki genetycznie uwarunkowanej – stwardnieniu guzowatym. Long-term, prospective study evaluating clinical and molecular biomarkers of epileptogenesis in a genetic model of epilepsy – tuberous sclerosis complex . Akronim badania: EPISTOP**

Główny badacz/koordynator projektu: prof. dr hab. n. med. Sergiusz Jóźwiak

Projekt międzynarodowy [nr rej. Komisji: 01/UE/13], z udziałem konsorcjum złożonego z 14 ośrodków z UE i USA, w tym 9 ośrodków klinicznych i 5 laboratoriów, w ramach 7PR Unii Europejskiej.

Komisja opiniowała następujące dokumenty:

1. Wniosek do Komisji Bioetycznej o zaopiniowanie zgłaszanego badania z dnia 28.12.2012
2. Streszczenie protokołu badania
3. Formularz Informacji dla Rodziców/Opiekunów Prawnych Pacjenta i Świadomej Zgody – wzory w języku polskim i angielskim (*Parents/Legal Guardians Participant Consent Form*)
4. Formularz Informacji dla Rodziców/Opiekunów Prawnych Pacjenta i Świadomej Zgody (wzór dla grupy kontrolnej) w języku polskim i angielskim (*Parents/Legal Guardians of Control Group Participant Consent Form*)
5. Formularz Informacji dla Rodziców/Opiekunów Prawnych Pacjenta i Świadomej Zgody (wzór dla pacjentów uczestniczących w badaniu bioptatów) w języku polskim i angielskim (*Parents/Legal Guardians of Participant Consent Form – Brain samples studies – epilepsy surgery patients*)
6. Formularz Informacji dla Rodziców/Opiekunów Prawnych Pacjenta i Świadomej Zgody (wzór) w języku polskim i angielskim (*Parents/ Legal Guardians of Participant Consent Form – Brain samples studies – post-mortem cases*)
7. Deklaracja Świadomej Zgody na badanie/a genetyczne – w języku polskim.

Protokół badania

Zgłoszenie badania dotyczy metody terapeutycznej, które będzie prowadzone z udziałem noworodków (0-27 dni) i niemowląt (28 dni – koniec 23 m.ż.) przez okres 5 lat. Projekt ma na celu badanie procesów epileptogenezy i możliwości jej modyfikacji w modelu padaczki genetycznie uwarunkowanej, jakim jest stwardnienie guzowate, w tym:

- analizę klinicznych i molekularnych biomarkerów epileptogenezy u dzieci z genetycznie uwarunkowana padaczką;
- ustalenie wpływu profilaktycznego (przed wystąpieniem napadów padaczkowych) leczenia przeciwpadaczkowego na rozwój dzieci, historię naturalną padaczki i jej lekooporność, a także na biomarkery kliniczne i molekularne padaczki,
- opracowanie testów diagnostycznych dla oceny ryzyka wystąpienia padaczki u dziecka z TSC,
- identyfikację nowych celów molekularnych dla nowych leków przeciwpadaczkowych i antyepileptogennych.

- 2 -

Badanie będzie się składało z trzech części:

1. prospektywnej oceny procesu epileptogenezy u dzieci z TSC
2. badania wpływu leczenia przeciwpadaczkowego na epileptogenezę, w zależności od momentu włączenia tego leczenia
3. badania markerów epileptogenezy w próbkach mózgu pobranych w czasie operacyjnego leczenia padaczki i w czasie autopsji u pacjentów z TSC.

W dwóch pierwszych częściach projektu uczestniczyć będą pacjenci, w trzeciej natomiast nie będzie elementu klinicznego.

Na podstawie przedłożonej dokumentacji, prezentacji prof. Sergiusza Jóźwiaka oraz wyniku dyskusji i tajnego głosowania Komisja Bioetyczna przy IPCZD wyraziła zgodę na przeprowadzenie projektu badawczego.

Skład i działanie Komisji zgodne z GCP oraz wymogami lokalnymi.
Lista członków Komisji biorących udział w posiedzeniu stanowi załącznik do niniejszego dokumentu.
Uwaga! Prof. K. Kotulska-Jóźwiak wyłączyła się i nie uczestniczyła w dyskusji i głosowaniu nad tym projektem.

PRZEWODNICZĄCA KOMISJI BIOETYCZNEJ
przy Instytucie „Pomnik-Centrum Zdrowia Dziecka"

Prof. dr hab. n. med. Joanna Pawłowska

Warszawa, dnia 31.01.2013 r.

KOMISJA BIOETYCZNA
przy Instytucie „Pomnik-Centrum Zdrowia Dziecka"
Al. Dzieci Polskich 20
04-730 Warszawa-Międzylesie
e-mail: komisja.bioetyczna@czd.pl
tel: (22) 815-75-71
fax: (22) 815-75-72

Lista obecności na posiedzeniu w dniu 23 stycznia 2013 roku

Lp.	Imię, nazwisko, zawód/specjalność, funkcja	Podpis
1	**PRZEWODNICZĄCA**<br>**Prof. dr hab. n. med. Joanna PAWŁOWSKA**<br>Lekarz (pediatra, transplantolog) – IPCZD	
2	**ZASTĘPCA PRZEWODNICZĄCEJ**<br>**Dr n. praw. Jerzy SŁYK**<br>Prawnik	
3	**HONOROWA PRZEWODNICZĄCA**<br>**Prof. dr hab. n. med. Maria GONCERZEWICZ**<br>Lekarz pediatra - emerytowany pierwszy Dyrektor CZD	nb
4	**Dr n. med. Ludmiła BACEWICZ**<br>Lekarz chirurg – IPCZD	
5	**Dr n. med. Joanna CIELECKA-KUSZYK**<br>Lekarz (pediatra, patomorfolog) - IPCZD	
6	**Prof. dr hab. n. med. Krystyna CHRZANOWSKA**<br>Lekarz genetyk - IPCZD	
7	**Dr n. hum. Maria DĄBROWSKA**<br>Psycholog Chrześcijańska Akademia Teologiczna	
8	**Dr hab. n. med. Katarzyna DZIERŻANOWSKA-FANGRAT, prof. nadzw.**<br>Lekarz mikrobiolog - IPCZD	
9	**Dr hab. n. med. Irena JANKOWSKA, prof. nadzw.**<br>Lekarz (pediatra, gastroenterolog, transplantolog) - IPCZD	
10	**Prof. dr hab. n. med. Wanda KAWALEC**<br>Lekarz (pediatra, kardiolog) – IPCZD	
11	**Dr hab. n. med. Katarzyna KOTULSKA-JÓŹWIAK, prof. nadzw.**<br>Lekarz neurolog - IPCZD	
12	**Dr n. med. Małgorzata ŁYSZKOWSKA**<br>Lekarz pediatra – IPCZD	
13	**Dr hab. n. med. Sylwester PROKURAT, prof. nadzw.**<br>Lekarz (pediatra, nefrolog, transplantolog) - IPCZD	
14	**Dr n. o zdrowiu Zofia SIENKIEWICZ**<br>Pielęgniarka, Zakład Pielęgniarstwa Społecznego WUM	nb
15	**Dr n. farm. Elżbieta WOJTASIK**<br>Farmakolog, Wydział Farmacji WUM	
16	**Ks. dr Arkadiusz ZAWISTOWSKI**<br>Teolog, Duszpasterstwo Służby Zdrowia	

Appendix 10

PTV
FONDAZIONE PTV
POLICLINICO TOR VERGATA

Comitato Etico Indipendente
(D.M. 08. 02. 2013 – D.G.R. 146/2013)

PROTOCOLLO DI STUDIO EPISTOP
REGISTRO SPERIMENTAZIONI 151/13

Roma, 5 dicembre 2013

Chiar.mo
Prof. Paolo Curatolo
U.O.C. Neuropsichiatria Infantile
Fondazione PTV
Policlinico Tor Vergata
SEDE

Notice is hereby given that, the Study **EPISTOP** "Long – term, prospective Study evaluating clinical and molecular Biomarkers of epileptogenesis in a genetic model of Epilepsy – Tuberosis Sclerosis Complex" was approved by Ethics Committee on 2nd December 2013.

Yours sincerely.

The Ethics Committee
Prof.ssa Maria Grazia Marciani

1

PTV
FONDAZIONE PTV
POLICLINICO TOR VERGATA

Comitato Etico Indipendente
(D.M. 8.2..2013 – D.G.R. 146/2013)

PRESIDENTE
Prof.ssa Rosaria ALVARO
(componente esterno)

Associato di Infermieristica Generale, Clinica e Pediatrica
Università degli Studi di Roma di "Tor Vergata"
"Rappresentante dell'area delle professioni sanitarie"

Prof. Massimo ANDREONI
(componente interno)

Ordinario di Malattie Infettive
Università degli Studi di Roma di "Tor Vergata"
Direttore U.O.C. Servizio di Malattie Infettive e Day Hospital
Fondazione PTV "Policlinico Tor Vergata"
"Clinico"

Prof. Ermenegildo ANSELMI
(componente esterno)

Aggregato di Medicina Legale
Dipartimento di Medicina sperimentale e Chirurgia
Università degli Studi di Roma di "Tor Vergata"
"Medico legale"

Prof.ssa Maria Luisa BARBACCIA
(componente esterno)

Ordinario di Farmacologia
Dipartimento di Medicina dei sistemi
Università degli Studi di Roma "Tor Vergata"
"Farmacologo"

Prof. Enrico BOLLERO
(componente interno)

Direttore Generale
Fondazione PTV Policlinico Tor Vergata
"Direttore Generale della Struttura sanitaria coinvolta nello Studio clinico"

Dott. Virgilio CALZINI
(componente esterno)

Medico Esperto in Medicina Generale
associato FIMMG
"Medico di medicina generale territoriale"

Dott.ssa Maria Grazia CELESTE
(componente interno)

Direttore di U.O.C. Farmacia aziendale
Fondazione PTV Policlinico Tor Vergata
"Farmacista del SSR"

Prof. Carlo CHIARAMONTE
(componente esterno)

Biostatistica e matematica attuariale
Docente a contratto di Lauree triennali della
Facoltà di Medicina e Chirurgia
Università degli Studi di Roma "Tor Vergata"
"Biostatistico"

2

Dr.ssa Patrizia DANIELI<br>(componente esterno)	Segretaria Regionale Associazione ANED<br>Associazione Nazionale Emodializzati<br>***"Rappresentante del volontariato per l'assistenza e/o dell'associazionismo di tutela dei pazienti"***
Prof. Claudio FRANCHINI<br>(componente esterno)	Ordinario di Diritto amministrativo<br>Dipartimento di Giurisprudenza<br>Università degli Studi di Roma "Tor Vergata"<br>***"Esperto in materia giuridica e assicurativa"***
Prof. Renato LAURO<br>(componente esterno)	Ordinario di Medicina Interna<br>Università degli Studi di Roma "Tor Vergata"<br>***"Clinico"***
Dott.ssa Marcella MARLETTA<br>(componente esterno)	Direttore Generale - Direzione Generale dei Dispositivi Medici del Servizio farmaceutico e della sicurezza delle cure<br>Ministero della Salute<br>***"Esperto in dispositivi medici"***
Dott. Corrado MASARACCHIA<br>(componente interno)	Farmacista afferente alla U.O.C. Farmacia clinica<br>Fondazione PTV Policlinico Tor Vergata<br>***"Farmacista del SSR"***
Dott.ssa Isabella MASTROBUONO<br>(componente interno)	Direttore Sanitario<br>Fondazione PTV Policlinico Tor Vergata<br>***"Direttore sanitario o suo sostituto permanente"***
Prof. Giuseppe NOVELLI<br>(componente interno)	Ordinario di Genetica Medica<br>Magnifico Rettore<br>Università degli Studi di Roma "Tor Vergata"<br>Direttore U.O.C. Genetica Medica<br>Fondazione PTV Policlinico Tor Vergata<br>***"Esperto di Genetica"***
Prof. Francesco PALLONE<br>(componente interno)	Ordinario di Gastroenterologia<br>Università degli Studi di Roma "Tor Vergata"<br>Direttore del Dipartimento di Medicina<br>Fondazione PTV Policlinico Tor Vergata<br>***"Clinico"***
Prof. Francesco PAONE<br>(componente interno)	Associato di Pediatria Generale e Specialistica<br>Università degli Studi di Roma "Tor Vergata"<br>Responsabile U.O.S.D. Pediatria e Gastroenterologia pediatrica<br>Fondazione PTV Policlinico Tor Vergata<br>***"Pediatra"***

3

Prof. Francesco ROMEO<br>(componente interno)	Ordinario di Cardiologia<br>Università degli Studi di Roma "Tor Vergata"<br>Direttore U.O.C. Cardiologia e Cardiologia interventistica<br>Fondazione PTV Policlinico Tor Vergata<br>***"Esperto clinico del settore, in relazione allo studio di nuove procedure tecniche diagnostiche e terapeutiche invasive e semi invasive"***
Prof. Claudio SARTEA<br>(componente esterno)	Aggregato di Filosofia del Diritto<br>Dipartimento di Giurisprudenza<br>Università degli Studi di Roma "Tor Vergata"<br>***"Esperto di Bioetica"***
Prof. Paolo SBRACCIA<br>(componente interno)	Ordinario di Scienze Tecniche Dietetiche Applicate<br>Università degli Studi di Roma "Tor Vergata"<br>Centro di Eccellenza Cura dell'Obesità e Scienze Dietetiche<br>Fondazione PTV Policlinico Tor Vergata<br>***"Esperto in nutrizione, in presenza di studi su prodotti alimentari"***
Dott. Alessandro SILI<br>(componente interno)	Coordinatore infermieristico<br>Responsabile U.O.C. Direzione Infermieristica e delle Professioni Sanitarie<br>Fondazione PTV Policlinico Tor Vergata<br>***"Rappresentante dell'area delle professioni sanitarie"***
Prof. Umberto TARANTINO<br>(componente interno)	Ordinario di Malattie Apparato Locomotore<br>Università degli Studi di Roma "Tor Vergata"<br>Direttore U.O.C Ortopedia e Traumatologia b<br>Fondazione PTV Policlinico Tor Vergata<br>***"Esperto qualificato, in relazione all'area medico – chirurgica oggetto dell'indagine con il dispositivo medico in studio"***

4

Appendix 11

FN MOTOL

ETICKÁ KOMISE PRO MULTICENTRICKÁ KLINICKÁ HODNOCENÍ
FAKULTNÍ NEMOCNICE V MOTOLE
Ethics Committee for Multi-Centric Clinical Trials of the University Hospital Motol
V úvalu 84, 150 06 Praha 5 ☎ 224 431 195 224 431 196
etickakomise@fnmotol.cz
www.fnmotol.cz

STANOVISKO ETICKÉ KOMISE K VÝZKUMNÉMU PROJEKTU
OPINION OF THE ETHICS COMMITTEE ON RESEARCH PROJECT

Název projektu / *Full Title of the Project* :

Dlouhodobá prospektivní studie hodnotící klinické a molekulární biomarkery epileptogeneze v genetickém modelu epilepsie – tuberózní sklerózy / *Epileptogenesis in a genetic model of epilepsy -Tuberous Sclerosis Complex* (EPISTOP)

ID číslo projektu / *Project No.* : **602391**

Zadavatel / *Sponsor:* **Výzva: FP7-HEALTH-2013- INNOVATION-1, Program: FP7**

Žadatel a hlavní zkoušející / *Applicant and Princpal Investigator:*
Doc. MUDr. Pavel Kršek, Ph.D., vedoucí elektrofyziologické laboratoře, Klinika dětské neurologie 2. LF UK a FN v Motole, V Úvalu 84, 150 06 Praha 5

EK vydává /*EC issues:* **souhlasné stanovisko / *favourable opinion***

Datum přijetí / *Date of Submission*: **9. 8. 2013**
Jednací č. / *Reference No.:* **EK-1205/13**
Datum jednání EK / *Date of EC Session:* **11. 9. 2013**

Etická komise prohlašuje, že byla ustavena a pracuje podle jednacího řádu v souladu se správnou klinickou praxí (GCP) a platnými předpisy / *The Ethics committee hereby declares that it was established and operates in accordance with its Rules of Procedure in compliance with Good Clinical Practice and valid legal regulations.*

**11. 9. 2013**	**MUDr. Vratislav Šmelhaus**	[signature]
Datum / *Date*	předseda/ *Chairman*	podpis předsedy EK / *Signature of Chairman*

Přiložen seznam členů komise (na druhé straně listu) / *List of the IEC members attached (in the other side)*

FAKULTNÍ NEMOCNICE V MOTOLE
150 06 Praha 5 - Motol, V Úvalu 84
Etická komise pro multicentrická hodnocení
tel.: 224 431 195 fax: 224 431 196
IČ: 00064203 DIČ: CZ00064203
80 / 810

Seznam členů Etické komise / *List of the IEC members*

**Jméno a příjmení** *Name and Surname*	**Muž / Žena** *Male / Female*	**Odbornost** *Occupation*	**Závislost** *Liability*	**Hlasoval** *Voted*
MUDr. Vratislav Šmelhaus<br>Předseda / *Chairman*	M	M.D.	☒	☒
Doc. MUDr. Jitka Zelenková, CSc.	F	M.D.	☒	☐
Prof. MUDr. Václav Chaloupecký, CSc.	M	M.D.	☒	☐
Doc. MUDr. Jiří Charvát, CSc.	M	M.D.	☒	☐
MUDr. Jiří Dušek, CSc.	M	M.D.	☒	☐
MUDr. Zdeněk Linke	M	M.D.	☒	☒
Doc. MUDr. Jan Zuna, Ph.D.	M	M.D.	☒	☒
Ing. Jana Hrdličková, Ph.D.	F	Chemist	☐	☒
JUDr. Radka Medková	F	Lawyer	☐	☒
Ivan Kazimour	M	Clark-laik	☒	☐
Prof. MUDr. Lidka Lisá, DrSc.	F	M.D.	☒	☐
Anna Dobešová	F	Clark-laik	☐	☒
PharmDr. Petr Horák	M	Pharmacist	☒	☐
Doc. MUDr. Jakub Hort, Ph.D.	M	M.D.	☒	☒
JUDr. Kateřina Královcová	F	Lawyer	☐	☒
Prof. MUDr. Michal Hrdlička, CSc.	M	M.D.	☒	☐

FAKULTNÍ NEMOCNICE V MOTOLE
150 06 Praha 5 - Motol, V Úvalu 84
Etická komise pro multicentrická hodnocení
tel.: 224 431 195 fax: 224 431 196
IČ: 00064203 DIČ: CZ00064203
810 / 08
