# Supplementary material for: Methodological insights from the EPISTOP trial to designing clinical trials in rare diseases—A secondary analysis of a randomized clinical trial
Source: PLoS One. 2024 Dec 3;19(12):e0312936. doi: 10.1371/journal.pone.0312936 (PMC11614242; doi:10.1371/journal.pone.0312936)
Supplement: S2 File — (PDF) [file pone.0312936.s002.pdf]

Reporting checklist for randomised trial.

Based on the CONSORT guidelines.

Instructions to authors

Complete this checklist by entering the page numbers from your manuscript where readers will find each of the items listed below.

Your article may not currently address all the items on the checklist. Please modify your text to include the missing information. If you are certain that an item does not apply, please write "n/a" and provide a short explanation.

Upload your completed checklist as an extra file when you submit to a journal.

In your methods section, say that you used the CONSORTreporting guidelines, and cite them as:

Schulz KF, Altman DG, Moher D, for the CONSORT Group. CONSORT 2010 Statement: updated guidelines for reporting parallel group randomised trials

		Reporting Item	comment/page
**Title and Abstract**			
Title	#1a	Identification as a randomized trial in the title.	"… - a secondary analysis of a randomized clinical trial" is added to the title, page 1
Abstract	#1b	Structured summary of trial design, methods, results, and conclusions	Abstract is structured accordingly, page 1
**Introduction**			
Background and objectives	#2a	Scientific background and explanation of rationale	Page 2 to 3

Background and objectives	#2b	Specific objectives or hypothesis	Page 3, line 71 ff.
**Methods**			
Trial design	#3a	Description of trial design (such as parallel, factorial) including allocation ratio.	Page 3, line 77 ff.
Trial design	#3b	Important changes to methods after trial commencement (such as eligibility criteria), with reasons	n.a.
Participants	#4a	Eligibility criteria for participants	“The study design an results are presented elsewhere [32]” page 3, line 79
Participants	#4b	Settings and locations where the data were collected	“The study design an results are presented elsewhere [32]” page 3, line 79
Interventions	#5	The experimental and control interventions for each group with sufficient details to allow replication, including how and when they were actually administered	“The study design an results are presented elsewhere [32]” page 3, line 79
Outcomes	#6a	Completely defined prespecified primary and secondary outcome measures, including how and when they were assessed	Page 3, line 97 ff.
Outcomes	#6b	Any changes to trial outcomes after the trial commenced, with reasons	n.a.

Sample size	#7a	How sample size was determined.	“The study design an results are presented elsewhere [32]” page 3, line 79
Sample size	#7b	When applicable, explanation of any interim analyses and stopping guidelines	n.a.
Randomization - Sequence generation	#8a	Method used to generate the random allocation sequence.	“The study design an results are presented elsewhere [32]” page 3, line 79
Randomization - Sequence generation	#8b	Type of randomization; details of any restriction (such as blocking and block size)	“The study design an results are presented elsewhere [32]” page 3, line 79
Randomization - Allocation concealment mechanism	#9	Mechanism used to implement the random allocation sequence (such as sequentially numbered containers), describing any steps taken to conceal the sequence until interventions were assigned	See study protocol
Randomization - Implementation	#10	Who generated the allocation sequence, who enrolled participants, and who assigned participants to interventions	See study protocol
Blinding	#11a	If done, who was blinded after assignment to interventions (for	“The study design an results are presented elsewhere [32]” page 3, line 79

		example, participants, care providers, those assessing outcomes) and how.	
Blinding	#11b	If relevant, description of the similarity of interventions	n.a.
Statistical methods	#12a	Statistical methods used to compare groups for primary and secondary outcomes	Page 4, line 121 ff., page 5, line 145 ff. primary outcome, page 5, line 162 ff. secondary outcome
Statistical methods	#12b	Methods for additional analyses, such as subgroup analyses and adjusted analyses	n.a.
**Results**			
Participant flow diagram (strongly recommended)	#13a	For each group, the numbers of participants who were randomly assigned, received intended treatment, and were analysed for the primary outcome	“The study design an results are presented elsewhere [32]” page 3, line 79, see figure 1 in [32]
Participant flow	#13b	For each group, losses and exclusions after randomization, together with reason	“The study design an results are presented elsewhere [32]” page 3, line 79, see figure 1 in [32]
Recruitment	#14a	Dates defining the periods of recruitment and follow-up	“The study design an results are presented elsewhere [32]” page 3, line 79
Recruitment	#14b	Why the trial ended or was stopped	n.a.
Baseline data	#15	A table showing baseline demographic and clinical characteristics for each	“The study design an results are presented elsewhere [32]” page 3, line 79, see table 1 in [32]

		group	
Numbers analysed	#16	For each group, number of participants (denominator) included in each analysis and whether the analysis was by original assigned groups	Page 6, table 2
Outcomes and estimation	#17a	For each primary and secondary outcome, results for each group, and the estimated effect size and its precision (such as 95% confidence interval)	Page 6, line 170 ff., page 7, line 187 ff.
Outcomes and estimation	#17b	For binary outcomes, presentation of both absolute and relative effect sizes is recommended	n.a.
Ancillary analyses	#18	Results of any other analyses performed, including subgroup analyses and adjusted analyses, distinguishing pre-specified from exploratory	n.a.
Harms	#19	All important harms or unintended effects in each group (For specific guidance see CONSORT for harms)	"The study design an results are presented elsewhere [32]" page 3, line 79, see table 5 in [32]
**Discussion**			
Limitations	#20	Trial limitations, addressing sources of potential bias, imprecision, and, if relevant, multiplicity of analyses	"The study design an results are presented elsewhere [32]" page 3, line 79

Generalisability	#21	Generalisability (external validity, applicability) of the trial findings	Page 10, line 267
Interpretation	#22	Interpretation consistent with results, balancing benefits and harms, and considering other relevant evidence	Page 9 to 11
Registration	#23	Registration number and name of trial registry	Page 3, line 80
**Other information**			
Protocol	#24	Where the full trial protocol can be accessed, if available	Attached for reviewers
Funding	#25	Sources of funding and other support (such as supply of drugs), role of funders	Page 11, line 315 ff.

None The CONSORT checklist is distributed under the terms of the Creative Commons Attribution License CC-BY. This checklist can be completed online using https://www.goodreports.org/, a tool made by the EQUATOR Network in collaboration with Penelope.ai
